# Acquired savolitinib resistance in non-small cell lung cancer arises via multiple mechanisms that converge on MET-independent mTOR and MYC activation

**DOI:** 10.18632/oncotarget.10859

**Published:** 2016-07-26

**Authors:** Ryan E. Henry, Evan R. Barry, Lillian Castriotta, Brendon Ladd, Aleksandra Markovets, Garry Beran, Yongxin Ren, Feng Zhou, Ammar Adam, Michael Zinda, Corinne Reimer, Weiguo Qing, Weiguo Su, Edwin Clark, Celina M. D'Cruz, Alwin G. Schuller

**Affiliations:** ^1^ AstraZeneca Pharmaceuticals PLC, Oncology Bioscience, Waltham, MA, USA; ^2^ AstraZeneca Pharmaceuticals PLC, Oncology Bioscience, Alderley Park, UK; ^3^ Hutchison Medi Pharma Ltd, Zhangjiang Hi-Tech Park, Shanghai, China

**Keywords:** MET, MYC, NSCLC, acquired resistance, savolitinib

## Abstract

Lung cancer is the most common cause of cancer death globally with a significant, unmet need for more efficacious treatments. The receptor tyrosine kinase MET has been implicated as an oncogene in numerous cancer subtypes, including non-small cell lung cancer (NSCLC). Here we explore the therapeutic potential of savolitinib (volitinib, AZD6094, HMPL-504), a potent and selective MET inhibitor, in NSCLC. *In vitro*, savolitinib inhibits MET phosphorylation with nanomolar potency, which correlates with blockade of PI3K/AKT and MAPK signaling as well as MYC down-regulation. *In vivo*, savolitinib causes inhibition of these pathways and significantly decreases growth of MET-dependent xenografts. To understand resistance mechanisms, we generated savolitinib resistance in *MET*-amplified NSCLC cell lines and analyzed individual clones. We found that upregulation of MYC and constitutive mTOR pathway activation is a conserved feature of resistant clones that can be overcome by knockdown of MYC or dual mTORC1/2 inhibition. Lastly, we demonstrate that mechanisms of resistance are heterogeneous, arising via a switch to EGFR dependence or by a requirement for PIM signaling. This work demonstrates the efficacy of savolitinib in NSCLC and characterizes acquired resistance, identifying both known and novel mechanisms that may inform combination strategies in the clinic.

## INTRODUCTION

The hepatocyte growth factor (HGF) receptor MET is a receptor tyrosine kinase (RTK) important in numerous biological functions ranging from embryonic development to tissue repair [1]. When HGF binds MET in normal tissues, several downstream pro-survival/pro-proliferation pathways are induced, including MAPK (MEK-ERK), PI3K/AKT and STAT3-among others [2]. A large body of evidence points to *MET* as a key oncogenic driver of several human cancers, including small cell (SCLC) and non-small cell lung cancers (NSCLC) [1]. Until the last decade, lung cancer was treated as a single, homogenous disease with static median survival rates of less than a year with chemotherapy. However, improved molecular diagnostics and an increased understanding of the molecular lesions driving lung cancers have facilitated better disease classification and the development of new treatments [3]. Accordingly, NSCLC cases are now classified based on both histology *and* genetic background, which has opened the door to personalized medicine approaches. Recent molecular characterization of patient samples demonstrates that NSCLC arises from alteration of a relatively small subset of genes [4–6], including *MET* copy number (CN) gain and exon 14 skipping, which together account for ~6.5% and 3.6% of driver mutations in lung adenocarcinoma (LUAD) and lung squamous cell carcinoma (LUSC) cases, respectively [4, 7]. Additional studies have detected *MET* CN gain in 2-22% of patients, while others have observed MET overexpression in a high percentage of patients [8].

Given the prevalence of MET aberrations across multiple cancer types, it is not surprising that MET has been a target of significant clinical interest and drug discovery efforts for several years. Two small-molecule multi-kinase inhibitors with MET inhibitory activity have been FDA approved: cabozantinib and crizotinib. The first of these, cabozantinib, is a multi-kinase inhibitor targeting RET, VEGFR2, KIT, TIE2, AXL and the FLT family of kinases in addition to MET [9] and was FDA-approved in November 2012 for clinical use in progressive metastatic medullary thyroid cancer. A year later, crizotinib, another multi-kinase inhibitor with activity against ALK, RON, ROS1 and MET [10] was granted FDA approval for ALK-positive metastatic NSCLC. However, the polypharmacology of multi-kinase inhibitors may limit their utility due to on- and off-target dose-limiting toxicities. Thus, there remains an unmet medical need for potent and highly selective MET inhibitors that may improve upon the ability of cabozantinib and crizotinib to inhibit MET signaling. To this end, more than a dozen clinical candidates, varying in mechanism of action and MET selectivity, have entered clinical trials in the last decade [11–12] ;1) antibodies that bind HGF and block receptor-ligand interaction, 2) antibodies that bind MET and prevent receptor-ligand interaction or receptor dimerization, and 3) small-molecule inhibitors that inhibit MET kinase activity [13]. The MET-binding antibodies ABT-700, LY2875358 and onartuzumab (MetMab) are in Phase I, I/II and I/II/III trials, respectively. Additionally, two HGF-binding antibodies—rilotumumab (AMG102) and ficlatuzumab (AV-299)—have entered trials. Rilotumumab reached Phase III trials before safety concerns halted its development in 2014. Several small-molecule MET inhibitors, including savolitinib, INC280, AMG337, LY2801653, SAR125844, MSC2156119J (EMD 1214063), JNJ-38877605 and PHA-665752 have progressed through Phase I and II trials in multiple cancer types; however, JNJ-38877605 trials were terminated due to renal toxicity/lack of a pharmacodynamic response, and development of PHA-665752 was stopped for undisclosed reasons. These clinical candidates have varying mechanisms of action which could result in differences in clinical utility. For example, HGF antibodies and the ligand-blocking, monovalent MET antibody onartuzumab may show benefit in ligand-dependent settings, whereas selective small-molecule MET inhibitors may show benefit in both ligand-dependent and ligand-independent settings [14–16].

Recently, the highly-selective and potent small-molecule MET inhibitor savolitinib (volitinib, AZD6094, HMPL-504) has been described [17], and work by our group and others has demonstrated the efficacy of savolitinib in preclinical models of gastric and papillary renal cell cancers [18–19]. While savolitinib is currently undergoing Phase I/II clinical testing, the therapeutic potential of savolitinib in lung cancers has not been determined. Here, we demonstrate MET dependency in select NSCLC models by targeting MET with savolitinib. *In vitro* and *in vivo*, we find that savolitinib inhibits MET, PI3K/AKT and MAPK signaling and downregulates MYC expression in NSCLC models. We further show that acquired savolitinib resistance occurs through reactivation of downstream kinase signaling and is driven by aberrant mTOR activation, MYC over-expression and context-specific reliance on EGFR signaling. Lastly, we uncover a novel role for PIM kinases in acquired savolitinib resistance and show that PIM inhibition restores savolitinib sensitivity both *in vitro* and *in vivo*. Together, this study demonstrates the preclinical efficacy of savolitinib in NSCLC and elucidates both known and novel mechanisms of acquired MET inhibitor resistance, identifying possible patient stratification and drug combination strategies to combat potential savolitinib resistance in the clinic.

## RESULTS

### Savolitinib potently inhibits MET activity and cell viability in pre-clinical NSCLC models *in vitro*

**Figure 1:**
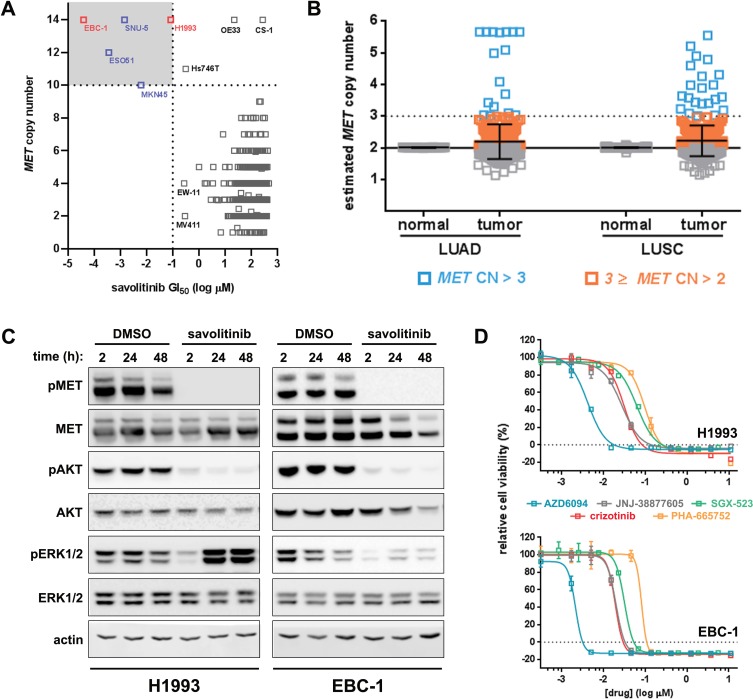
Savolitinib sensitivity in NSCLC cell lines occurs predominately in the *MET*-amplified setting **A.** Sanger cell line screening for savolitinib sensitivity. Cell line names are shown for those lines with ≥10 copies of *MET*. Cell lines with savolitinib GI_50_ values of ≤100 nM and *MET* CN of ≥10 are highlighted in the upper-left quandrant. **B.** *MET* copy number (CN) analysis of lung adenocarcinoma (LUAD), lung squamous cell carcinoma (LUSC) and normal tissue from LUAD/LUSC patients (TCGA dataset). *MET* CN gain is defined as high-level (open blue squares) or low-level (open orange squares). Samples with *MET* CN ≤ 2 are shown as open grey squares. *MET* CN data shown are the mean ± S.D. **C.** immunoblot analysis of H1993 and EBC-1 cells treated with 100 nM savolitinib for the indicated times. **D.** MET inhibitor GI_50_ determination in H1993 and EBC-1 cells treated as indicated for five days. Data shown are the mean ± S.D. normalized to vehicle control.

*MET* CN gain leading to MET dependence can predict MET small-molecule inhibitor sensitivity of tumor cells [20]. In order to select appropriate models for *in vitro* interrogation, we determined savolitinib GI_50_ values for more than 900 cell lines present in the Sanger Cell Line Panel [21–22], 111 of which represent NSCLC. *In vitro*, we found that those cell lines most sensitive to savolitinib were highly-amplified for *MET*, harboring ten or more copies (Figure 1A, Supplementary Figure S1A). To assess the frequency of *MET* CN gain in actual LUAD and LUSC patients, we undertook a bioinformatic analysis of clinical samples from TCGA datasets. We analyzed normal and tumor tissue from 506 LUAD and 501 LUSC patients and found low-level *MET* gain (CN >2 but ≤3) in 1.38% and 2.98% of LUAD and LUSC tumors, respectively, while high-level *MET* gain (CN >3) was present in 1.97% of LUAD and 1.39% of LUSC samples (Figure 1B). We therefore estimate the overall rate of *MET* CN gain to be ~3.35% and ~4.37% in LUAD and LUSC patients, respectively.

Among the five cell lines with savolitinib GI_50_ values less than 100 nM, H1993 (LUAD) and EBC-1 (LUSC) showed nanomolar savolitinib sensitivity, in agreement with previous work [20, 23], and were selected for further study. To determine the effects of savolitinib on the MET signaling pathway, H1993 and EBC-1 cells were treated with 100 nM savolitinib over a time course and subjected to immunoblot analysis. Treatment with savolitinib potently inhibited MET phosphorylation at Y1234/1235 at all time points tested (Figure 1C). Downstream of MET, AKT signaling was strongly inhibited by savolitinib for the duration of treatment, whereas MAPK signaling, as represented by ERK1/2 phosphorylation, was inhibited at two hours after drug treatment but was reactivated in both cell lines in a MET-independent manner (Figure 1C). To determine the relative potency of savolitinib *versus* other MET inhibitors, we performed a five-day proliferation assay comparing savolitinib to the selective MET inhibitors PHA-665752, JNJ-38877605 and SGX-523 as well as crizotinib, a multi-kinase inhibitor approved for clinical use. Savolitinib was more potent than all other molecules tested, with GI_50_ values of 4.20 nM and 2.14 nM in H1993 and EBC-1 cells, respectively (Figure 1D). Importantly, savolitinib did not affect the viability of an NSCLC line (Supplementary Figure S1B, C) or gastric cancer models lacking *MET* CN gain [18–19]. Lastly, to determine whether dose-dependent inhibition of cell viability by savolitinib correlates with the extent of pMET inhibition, H1993 and EBC-1 cells were treated with a dilution series of savolitinib for two hours followed by immunoblot analysis and quantitation of pMET expression. Indeed, when pMET inhibition was analyzed by densitometry, the IC_50_ values were 4.12 nM and 4.25 nM for H1993 and EBC-1, respectively, in close agreement with the GI_50_ values for both cell lines (Supplementary Figure S1D, E). Likewise, when we repeated the experiment with a less-potent MET inhibitor (crizotinib), IC_50_ values for MET inhibition were right-shifted to 77.4 nM and 17.95 nM for H1993 and EBC-1 cells, respectively (Supplementary Figure S1D, E). In sum, savolitinib potently inhibits MET signaling and reduces the viability of MET-dependent NSCLC lines in a dose-dependent manner *in vitro*.

### Savolitinib is efficacious in cell line- and patient-derived xenograft NSCLC models

**Figure 2:**
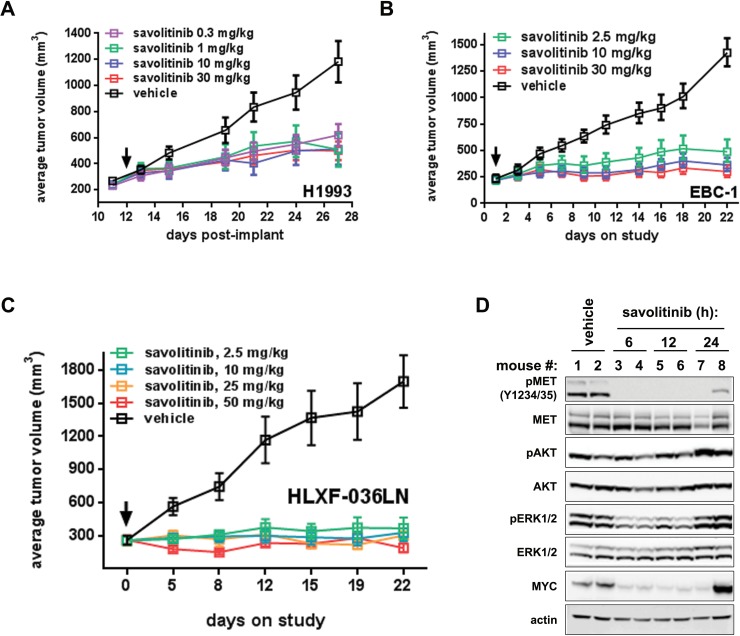
Savolitinib inhibits MET activity and tumor growth in NSCLC xenograft models **A.**-**B.**, efficacy in H1993 (A) and EBC-1 (B) xenografts treated once daily as indicated. **C.**, efficacy in the HLXF-036LN PDX model treated once daily as indicated. **D.**, pharmacodynamic analysis of HLXF-036LN tumor-bearing mice treated with vehicle or 50.0 mg/kg savolitinib for the indicated times. Data shown are from two independent tumors per time point.

We next tested savolitinib efficacy *in vivo* using H1993 and EBC-1 tumor xenografts. Savolitinib treatment led to marked decreases in tumor growth relative to vehicle controls in both models over the full dose ranges tested (Figure 2A, 2B), with savolitinib achieving a maximal response at doses as low as 0.3 mg/kg and 2.5 mg/kg in H1993 and EBC-1 tumors, respectively. In addition to the savolitinib groups shown in Figure 2A, we also assessed crizotinib efficacy at 1, 3 and 30 mg/kg given once daily in the H1993 tumor model, allowing us to extend our *in vitro* comparison of savolitinib and crizotinib to the *in vivo* setting. Interestingly, while savolitinib did not inhibit H1993 tumor growth in a dose-responsive manner over the dose ranges tested, crizotinib did demonstrate a dose response, achieving ~30% ± 15.81% (s.e.m.), ~31% ± 10.49% or ~61% ± 6.82% (s.e.m.) TGI at 1, 3 and 30 mg/kg doses, respectively. (Supplementary Figure S2A). In sum, these results demonstrate that savolitinib effectively reduces the growth of MET-dependent NSCLC cell line models *in vivo* and reaches its maximum achievable efficacy in H1993 at lower doses than the less-selective MET inhibitor crizotinib.

We further conducted a pharmacodynamic analysis of H1993 tumors from the efficacy study shown in Figures 2A and S2A. On the last day of treatment, tumors were harvested at three and eight hours following a final dose with vehicle or 1, 3 or 30 mg/kg savolitinib or crizotinib. Immunoblot analysis of multiple biomarkers from savolitinib-treated tumors revealed complete inhibition of MET phosphorylation with concomitant inhibition of ERK1/2, AKT and S6 phosphorylation, as well as downregulation of MYC three hours after dosing (Supplementary Figure S2B). Analysis of tumors eight hours post-treatment shows that MET activation, as well as the activation of downstream kinases, begins to return to baseline levels, with the degree of recovery inversely correlating with savolitinib dose (Supplementary Figure S2C). Interestingly, 1 and 3 mg/kg crizotinib treatment resulted in little to no modulation of MET phosphorylation or downstream kinases at three or eight hours after dosing (Supplementary Figure S2B, C), whereas pharmacodynamic changes in the 30 mg/kg crizotinib-treated tumors more closely mirrored those seen in tumors from savolitinib-treated mice (Supplementary Figure S2B, C).

Lastly, we tested savolitinib *in vivo* activity in HLXF-036LN, a patient-derived xenograft (PDX) derived from a NSCLC lymph-node metastasis. The HLXF-036LN tumor model harbors 3.7 copies of *MET* and displays robust basal MET phosphorylation, indicating that MET is activated in this model (Figure 2D and data not shown). Savolitinib induced regressions (28%) at 50 mg/kg (Figure 2C). Immunoblotting of HLXF-036LN lysates showed that phospho-MET was inhibited for 12 hours in all animals and for 24 hours in one animal after a single 50 mg/kg dose (Figure 2D). Interestingly, we also observed a robust decrease in MYC levels, closely correlating with phospho-MET inhibition (Figure 2D) and consistent with results obtained in the H1993 and EBC-1 xenograft studies. Together, these data demonstrate that savolitinib inhibits MET *in vivo* to drive tumor growth inhibition or regressions in several NSCLC xenograft models.

### *In vitro* savolitinib resistance is stable and occurs without MET reactivation or mutation

**Figure 3:**
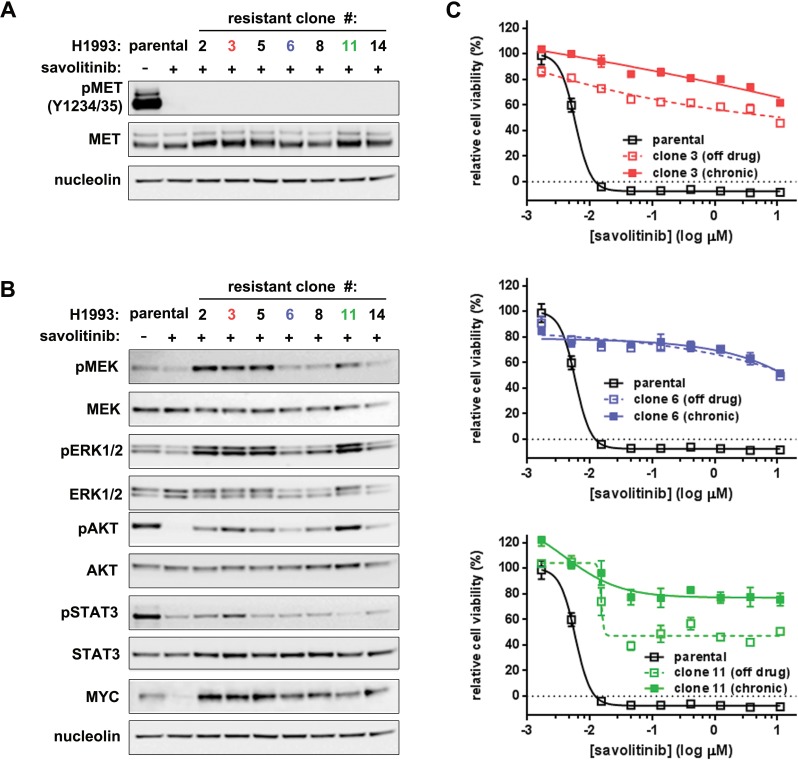
Durable savolitinib resistance is associated with MYC upregulation without *MET* reactivation **A.**-**B.**, immunoblot analysis of parental H1993 cells and seven savolitinib-resistant clones. Levels of total MET (MET) and activated MET (pMET; p-Y1234/Y1235) (A) and downstream signaling molecules (B) were measured in cells treated with 2 μM savolitinib for four hours. **C.**, assessing resistance stability over time. The indicated clones were cultured in the presence or absence of 2 μM savolitinib for five weeks (solid and open colored squares, respectively) followed by treatment with a dose range of savolitinib for five days and subsequent cell viability measurement. Parental cells serve as a reference for savolitinib sensitivity (open black squares).

Having demonstrated savolitinib efficacy in preclinical NSCLC models, we next sought to identify possible mechanisms of acquired savolitinib resistance. H1993 cells were treated with sequentially higher concentrations of savolitinib, starting below the GI_50_, until cells grew in 2.0 μM compound as a polyclonal population; seven clonal subpopulations were subsequently isolated. Interestingly, each savolitinib-resistant clone possessed a different morphology than the parental cells, some with a fibroblast-like appearance (Supplementary Figure S3A). Surprisingly, phospho-MET inhibition by savolitinib was indistinguishable between parental cells and clones (Figure 3A), suggesting the absence of activating *MET* mutations or *MET* CN gains, which we confirmed by exome sequencing (Supplementary Figure S3B, C and NCBI Sequence Read Archive SubmissionID SUB1084746).

Signaling activity downstream of MET is variable between clones. Notably, after savolitinib treatment for 24 hours, AKT was reactivated in all clones while increased MEK/ERK activity was present in four of seven clones (Figure 3B). Interestingly, cMYC (hereafter referred to as MYC) protein levels dropped significantly in parental savolitinib-treated H1993 cells, but MYC down-regulation was uncoupled from MET inhibition in all clones (Figure 3B). Exome sequencing revealed that parental H1993 cells and clones are diploid for *MYC* (Supplementary Figure S3D), ruling out *MYC* amplification as a mechanism of savolitinib resistance, a phenomenon that has been shown to drive resistance to other targeted therapies [24]. On the basis of downstream signaling status, savolitinib-resistant H1993 clones were classified into three sub-types; one clone from each sub-type — H1993 clones 3, 6 and 11 (hereafter individually referred to in the text as clone 3, clone 6 and clone 11 or collectively as ‘clones’) — was selected for further study. To assess resistance stability, clones were passaged in the presence or absence of 2 μM savolitinib for five weeks, followed by re-exposure to savolitinib. All three clones retained resistance to savolitinib, as well as several other MET inhibitors (Figure 3C and Supplementary Figure S3E). Together, these data demonstrate that acquired savolitinib resistance is stable and associated with the uncoupling of MET activity from downstream kinase signaling and MYC expression.

### Savolitinib resistance in NSCLC is partially driven by MYC overexpression

**Figure 4:**
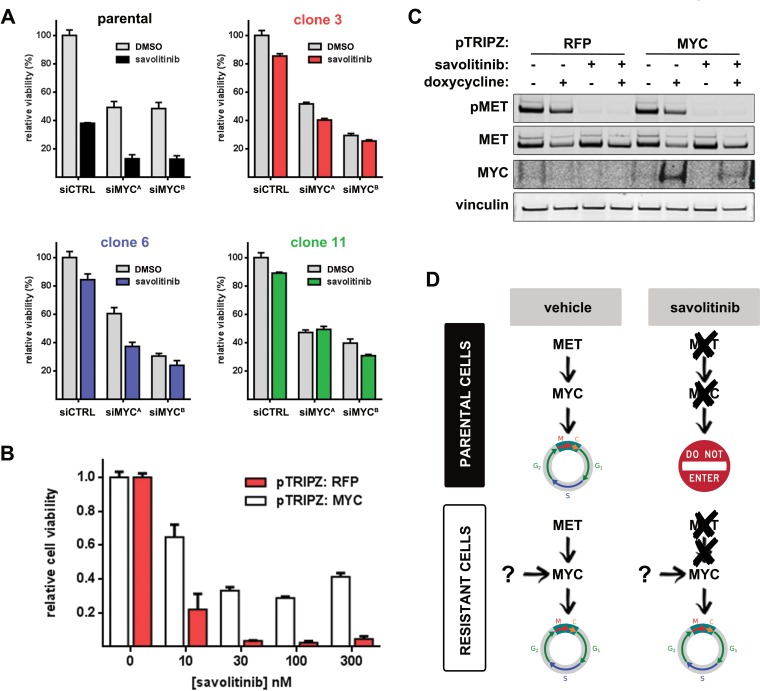
Sustained MYC expression is required for savolitinib resistance **A.**, cell viability following MYC knockdown ± savolitinib treatment. Cells were transfected with control siRNA (siSCR) or MYC-targeting siRNAs (siMYC^A^, siMYC^B^) and treated with vehicle or 100 nM savolitinib for 72 hours. Data shown are the mean ± S.D. of three replicates. **B.**, viability of parental H1993 cells stably expressing a doxycycline-inducible RFP (negative control) or human MYC. Cell viability was assessed after 24 hours of doxycycline treatment followed by vehicle or savolitinib treatment for 24 hours. **C.**, immunoblot validation of MET inhibition and MYC overexpression for a single savolitinib dose (100 nM). Vinculin serves as a loading control. **D.**, a schematic consistent with our data summarizing the putative role of MYC deregulation in savolitinib resistance.

To determine whether deregulated MYC expression functionally promotes savolitinib resistance, we first used siRNA to knock down MYC in parental and resistant H1993 cells and treated with vehicle or 100 nM savolitinib for three days followed by cell viability measurements. Interestingly, viability was MYC-dependent irrespective of savolitinib treatment in both parental cells and clones (Figure 4A; Supplementary Figure S4A). MYC knockdown alone reduced viability of parental cells to a similar extent as savolitinib treatment, suggesting that MYC down-regulation in parental cells drives savolitinib efficacy to a significant degree and that sustained MYC expression in savolitinib-treated resistant clones may be a critical mediator of savolitinib resistance.

To test the hypothesis that uncoupling of MYC expression from MET signaling —thereby resulting in sustained MYC expression — drives savolitinib resistance, we generated parental H1993 cells stably expressing doxycycline-inducible red fluorescent protein (RFP) or human MYC and treated each with a range of savolitinib doses following RFP/MYC induction. Strikingly, MYC — but not RFP — overexpression partially rescued the effects of savolitinib treatment, even at 300 nM (Figure 4B); MYC overexpression and MET inhibition were confirmed by immunoblot (Figure 4C). We extended these findings by generating and characterizing a savolitinib-resistant EBC-1 cell population. Consistent with our findings in H1993 cells, savolitinib potently inhibited MET phosphorylation in resistant EBC-1 cells while AKT and ERK phosphorylation levels remained high (Supplementary Figure S4B). Importantly, basal MYC protein levels were also upregulated and unaffected by savolitinib exposure (Supplementary Figure S4B). Attenuation of MYC expression strongly inhibited resistant EBC-1 cell viability proportional to the degree of MYC knockdown (Supplementary Figure S4C, D), suggesting that MYC is also required for savolitinib resistance in a LUSC model. A schematic illustrating the role of MYC in acquired savolitinib resistance is presented in Figure 4D.

### EGFR inhibition is synthetically lethal with savolitinib treatment in a clone-specific manner

**Figure 5:**
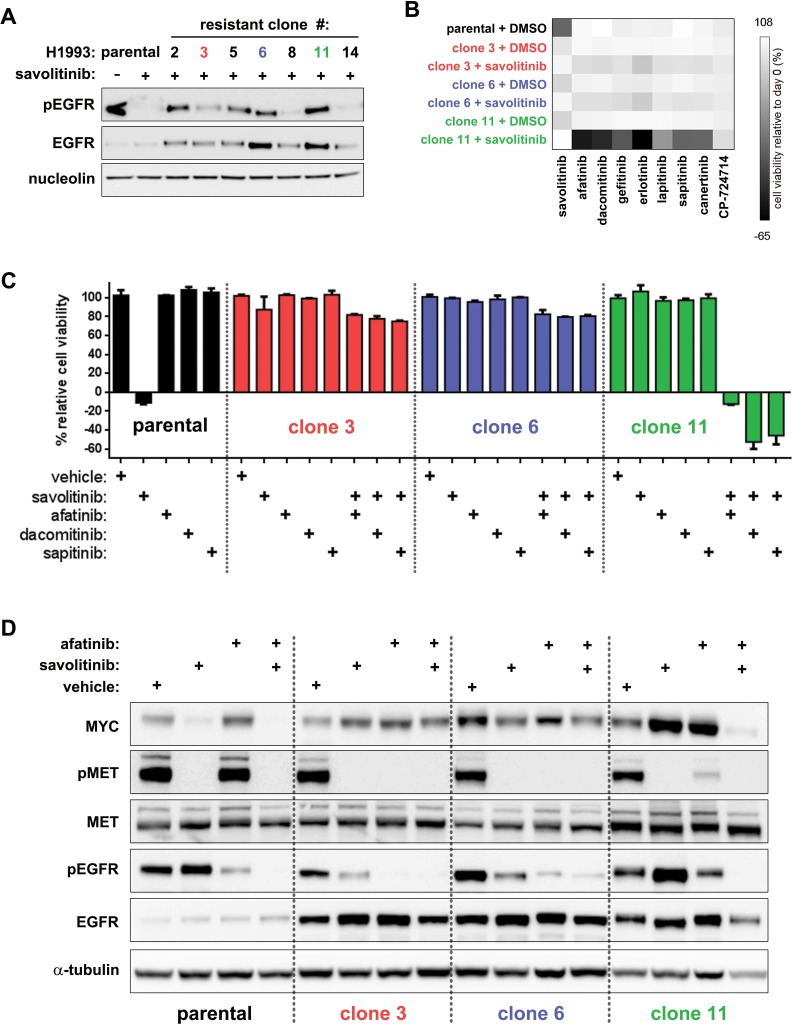
EGFR inhibition is synthetically lethal with savolitinib in a clone-specific manner **A.**, immunoblot analysis of total and activated EGFR (pEGFR) levels in parental H1993 cells and clones treated with 2 μM savolitinib for four hours. **B.**, cell viability heatmap for parental H1993 cells and clones treated with the indicated EGFR inhibitors (137 nM) for five days. Clones were seeded in either 0.1% DMSO or 100 nM savolitinib 24 hours prior to treatment. **C.**, five-day viability assay for parental H1993 cells treated with single-agent savolitinib or the indicated EGFR inhibitors. Clones were treated with each EGFR inhibitor alone or in combination with 100 nM savolitinib. Data shown are the mean ± s.d. cell viabilities determined at 412 nM of each EGFR inhibitor, which is a concentration near the middle of the nine-point dose response curve tested. **D.**, immunoblot analysis of parental H1993 cells and clones treated with 0.1% DMSO or 100 nM savolitinib, afatinib or the combination (100 nM each) for eight hours.

Previous studies of acquired MET inhibitor resistance in several tumor types, including NSCLC, suggest that *de novo* dependence on EGFR signaling is a common means to circumvent MET inhibition [25–26]. We therefore hypothesized that a switch to EGFR dependence in H1993 clones sustains MYC expression during savolitinib exposure. Analysis of savolitinib-treated clones indicates that several expressed more total EGFR and activated (pEGFR, Y1068) protein relative to parental cells (Figure 5A); clone 6 and clone 11 express significantly higher levels whereas clone 3 shows a more modest increase. NGS analysis revealed that parental and resistant cells were diploid for *EGFR*, thus, enhanced EGFR expression in resistant clones was independent of *EGFR* amplification (Supplementary Figure S5A).

The functional relevance of EGFR overexpression was tested by inhibiting EGFR family proteins in the presence or absence of savolitinib. Clones were plated in either 0.1% DMSO or 100 nM savolitinib 24 hours prior to screening of eight small-molecule EGFR inhibitors, each covering a dose range between ~11.1 μM and ~1.7 nM. Parental cells served as a control and were plated in 0.1% DMSO only. Surprisingly, only clone 11 was dependent on EGFR activity, and only in combination with savolitinib, suggesting clone 11 becomes EGFR-dependent only upon MET inhibition (Figure 5B).

To confirm our screen results, we determined the viability of parental H1993 cells and clones treated with afatinib, dacomitinib or sapitinib (AZD8931, [27]). All EGFR inhibitors failed to reduce viability when given alone and only modestly affected viability of clone 3 and clone 6 when combined with savolitinib. However, savolitinib combined with afatinib, dacomitinib or sapitinib induced cell death in clone 11 (Figure 5C), consistent with our preliminary findings. To assess whether EGFR inhibition alone or in combination with savolitinib could affect MYC expression, resistant cells were treated with 100 nM afatinib +/− 100 nM savolitinib for eight hours followed by immunoblot analysis. Indeed, only combined EGFR/MET blockade concomitantly inhibited EGFR activation and blocked MYC expression in clone 11 (Figure 5D). Together, these data demonstrate that a switch to EGFR signaling maintains MYC levels and confers savolitinib resistance in a clone-specific manner.

To determine if EGFR activation is a more general mechanism of savolitinib resistance in NSCLC, we assessed EGFR activation and EGFR inhibitor sensitivity in savolitinib-resistant EBC-1 cells. Indeed, resistant EBC-1 cells show increased basal EGFR phosphorylation that is unchanged following treatment with 100 nM savolitinib for up to 24-48 hours (Supplementary Figure S5B), suggesting that EGFR drives survival and proliferation in savolitinib-treated resistant EBC-1 cells. Indeed, whereas parental EBC-1 cells are resistant to each of three different EGFR family inhibitors (Supplementary Figure S5C), resistant EBC-1 cells are highly sensitive to EGFR inhibitors when combined with savolitinib but are resistant to EGFR inhibitor monotherapy (Supplementary Figure S5D-F). Thus, a switch to EGFR-dependent survival and proliferation may be a conserved mechanism of savolitinib resistance across NSCLC subtypes.

### Dual mTORC1/2 inhibitors resensitize resistant cells to savolitinib

**Figure 6:**
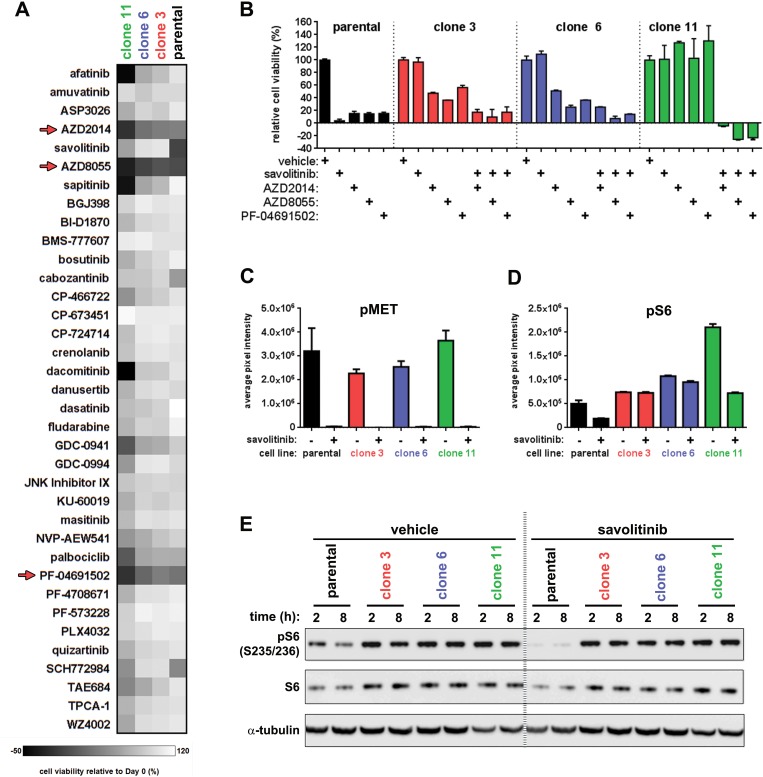
A chemical screen reveals that dual mTORC1/2 inhibitors resensitize resistant cells to savolitinib **A.**, cell viability heatmap for parental H1993 cells and clones treated with the indicated compounds for five days. Cells were treated with a nine-point dose response curve of each drug ranging from 11.1 μM to 1.7 nM. Parental H1993 cells and clones were seeded in media containing 0.1% DMSO or 100 nM savolitinib, respectively. Dual mTORC1/2 inhibitors are indicated with red arrows. For clarity, the viability data from only one of the nine doses tested for each drug (137 nM) are shown. **B.**, five-day viability assay for parental H1993 cells treated with single-agent savolitinib or the indicated mTORC1/2 inhibitors. Clones were treated with each mTORC1/2 inhibitor alone or in combination with 100 nM savolitinib. Data shown are the mean ± s.d. cell viabilities determined at 412 nM of each mTORC1/2 inhibitor, which is a concentration near the middle of the nine-point dose response curve tested. **C.**-**D.**, phospho-protein array densitometry. Data shown are the mean ± S.D. of duplicate spots on the array. pMET signal represents pan-tyrosine phosphorylation of MET; pS6 signal represents phosphorylation at S235/236. **E**. confirmation of phospho-protein array results revealing differential modulation of pS6 (S235/236) levels between parental H1993 cells and resistant clones following MET inhibition. Cells were treated with 0.1% DMSO or 100 nM savolitinib for two or eight hours and lysates analyzed by immunoblot. α-tubulin serves as a loading control.

To identify EGFR-independent mechanisms of resistance, we screened a panel of 36 small-molecules targeting multiple RTKs and downstream signaling kinases/targets. Data for a single concentration of each compound are shown in Figure 6A. Savolitinib, dacomitinib, sapitinib and afatinib were included as positive controls. Overall, most compounds tested had little effect on parental or resistant cell viability. However, three dual mTORC1/2 inhibitors — AZD2014, AZD8055 and the less-selective, dual mTOR/PI3K inhibitor PF-04691502 [28] — reduced the viability of all four cell lines (Figure 6A).

Screen results were confirmed by treating resistant clones with a nine-point dose response curve of AZD2014, AZD8055 or PF-04691502, ranging from ~11.1 μM to ~1.7nM, in the presence or absence of 100 nM savolitinib, followed by viability measurements. Parental cells were treated with a nine-point dose curve of savolitinib or each of the mTORC1/2 inhibitors as single-agents only. All mTORC1/2 inhibitors showed single-agent activity in parental cells and in clone 3 and clone 6, reducing viability by ~90% and ~50-70%, respectively. In contrast, mTORC1/2 inhibitors were inactive in clone 11 (Figure 6B). Combination of mTORC1/2 inhibitors with savolitinib further reduced viability of clone 3 and clone 6. Interestingly, the combination was synthetically lethal in clone 11 (Figure 6B). Similarly, all three mTORC1/2 inhibitors showed single-agent activity in parental EBC-1 cells as well as in the savolitinib-resistant EBC-1 cell population. Furthermore, combination of savolitinib (100 nM) with either AZD8055, AZD2014 or PF-04691502 resulted in synergistic induction of growth arrest or cell death at mTORC1/2 inhibitor concentrations above ~400 nM (Supplementary Figure S6A-D).

Concurrent with small-molecule screening, we measured the phosphorylation status of a focused target panel using a phospho-protein array. We plated parental H1993 cells and clones in drug-free medium for 24 hours, treated with 0.1% DMSO or 2 μM savolitinib for four hours and applied the lysates to the arrays. MET phosphorylation was strongly downregulated in all four cell lines following savolitinib treatment (Figure 6C and Supplementary Figure S7A). Interestingly, savolitinib downregulated pS6 levels in parental cells, whereas pS6 remained elevated in clones relative to parental levels irrespective of savolitinib treatment (Figure 6D and Supplementary Figure S7B); array results were subsequently confirmed by immunoblot analysis (Figure 6E). Given that S6 is principally phosphorylated by mTORC1 via activation of the S6 kinases [29], we treated parental cells and clones with the mTORC1-specific inhibitor RAD001 (everolimus) and measured cell viability. As a monotherapy, RAD001 reduced the viability of parental cells, clone 3 and clone 6 by ~30% but had no effect on clone 11 viability (Supplementary Figure S7C). Interestingly, combination of RAD001 with savolitinib failed to increase the effects of RAD001 alone on clone 3 and clone 6 viability, but synergized to produce a ~50% reduction in viability of clone 11, suggesting that clone 11 utilizes an mTORC1-independent survival pathway when MET is active but becomes mTORC1-dependent in the presence of savolitinib.

Further analysis of the AKT/mTOR/S6 axis revealed additional clone-specific differences in the activation state of this pathway. First, we found that savolitinib treatment of parental H1993 and clone 11 cells strongly reduced phosphorylation of AKT and the direct AKT substrate PRAS40, a negative regulator of the mTORC1 kinase complex [30] (Supplementary Figure S7D). Thus, inhibition of AKT and PRAS40 phosphorylation correlates with pS6 downregulation and reduced viability of parental, but not clone 11, cells. Given these findings and the pharmacological data demonstrating the resistance of clone 11 cells to single-agent mTORC1/2 inhibitors, we conclude that clone 11 cells utilize an mTOR-independent mechanism to maintain viability in the absence of savolitinib.

As further evidence of clone-specific savolitinib resistance mechanisms, we found that neither AKT nor PRAS40 phosphorylation were reduced by savolitinib treatment of clone 3 and clone 6 cells (Supplementary Figure S7D), and both cell lines are sensitive to single-agent mTORC1/2 inhibitors irrespective of savolitinib treatment (Figure 6B). Given this, we tested the effect of inhibitors targeting the key mTOR-activating kinases AKT [31–32] and ERK [33]. Resistant clones were treated with a nine-point dose response curve of AZD5363 (AKT inhibitor) or SCH772984 (ERK inhibitor) for five days in the presence or absence of 100 nM savolitinib and assessed for viability. Again, parental cells were treated with a nine-point dose curve of each single-agent only. Interestingly, neither AKT nor ERK inhibition significantly affected the viability of clone 3 or clone 6 cells, either as single agents or in combination with savolitinib (Supplementary Figure S7E). Taken together, these data suggest that non-canonical mechanism(s) of mTOR pathway activation, examples of which have been described previously [34–35], may drive savolitinib resistance in all three resistant clones studied.

### PIM inhibition restores savolitinib sensitivity in a clone-specific manner

**Figure 7:**
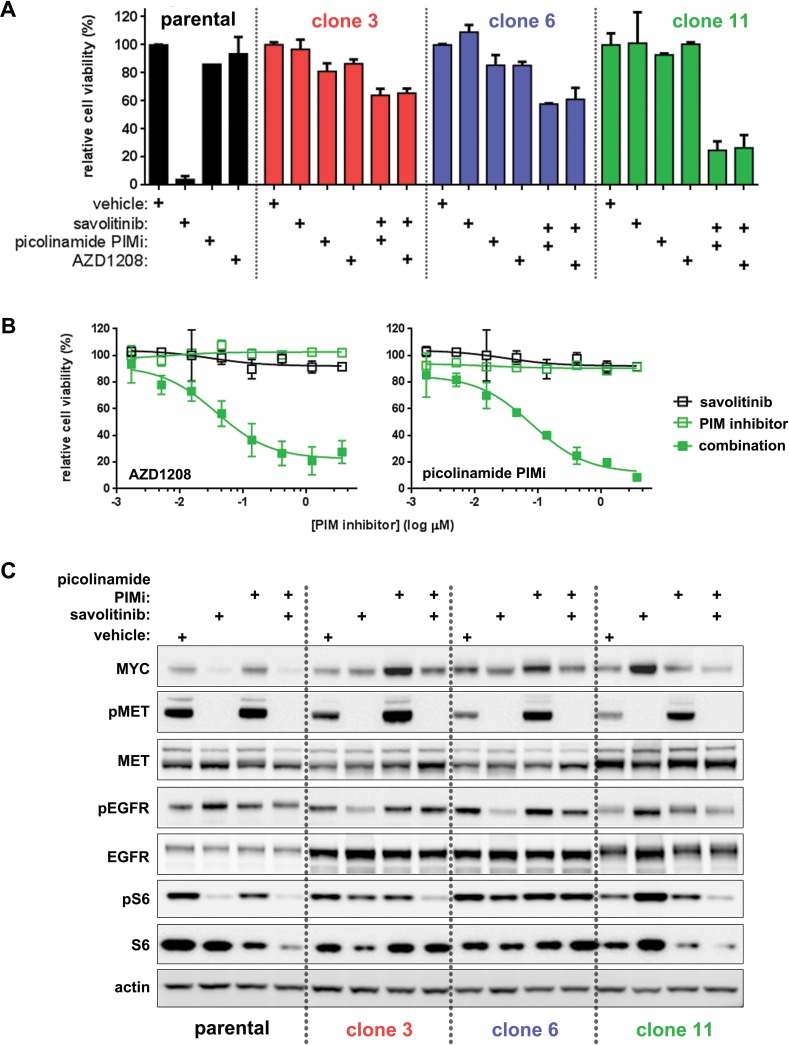
Clone-specific restoration of savolitinib sensitivity by PIM kinase inhibitors **A.**, five-day viability assay for parental H1993 cells treated with single-agent savolitinib or the indicated PIM inhibitors. Clones were treated with each PIM inhibitor alone or in combination with 100 nM savolitinib. Data shown are the mean ± s.d. cell viabilities determined at 412 nM of each PIM inhibitor, which is a concentration near the middle of the nine-point dose response curve tested. **B.**, full dose response plots of cell viability shown in panel A for clone 11 cells treated with a dose range of savolitinib alone (open black squares), each PIM inhibitor alone (open green squares) or combination of 100 nM savolitinib with a dose range of each PIM inhibitor (solid green squares). **C.**, immunoblot analysis of MYC, pEGFR and pS6 in parental H1993 cells and clone 3, clone 6 and clone 11 cells treated with vehicle, 100 nM savolitinib, 100 nM picolinamide PIMi or the combination (100 nM each) for eight hours.

Using a candidate approach to identify non-canonical mTOR activators, we tested PIM kinase inhibitors. The PIM family can promote tumorigenesis [36], and PIM-1 has been shown to stimulate mTOR signaling through PRAS40 inhibition [37]. Savolitinib-resistant cells were treated with two unique pan-PIM inhibitors — a picolinamide pan-PIM inhibitor [38–39] related to LGB-321 [40] — and AZD1208 [41] over the standard nine-point dose response range in the presence or absence of 100 nM savolitinib. Viability assays revealed that pan-PIM inhibition alone had no effect on parental H1993cells or clones (Figure 7A). Interestingly, combined PIM/MET inhibition potently synergized to inhibit cell growth in clone 11, but only mildly affected clone 3 and clone 6 (Figure 7A, 7B). Furthermore, PIM inhibition reversed savolitinib-induced pEGFR and MYC upregulation and downregulated total and phospho-S6 levels in clone 11 (Figure 7C). Similar to results found in H1993 cells, neither the parental nor the savolitinib-resistant EBC-1 cells respond to single-agent treatment with AZD1208; however, resistant EBC-1 cells become sensitive to AZD1208 in the presence of savolitinib (Supplementary Figure S8). Taken together, these results demonstrate that savolitinib resistance can be mediated by *de novo* dependence on PIM kinase signaling, a novel mechanistic finding in the context of MET inhibitor resistance. Lastly, during preparation of our manuscript, An et al. reported that PIM signaling can mediate MET inhibitor resistance by regulating BCL2 translation [42], corroborating our discovery of a novel role for PIM kinases in mediating MET inhibitor resistance.

### The H1993 clone 11 model maintains savolitinib resistance and clone-specific signaling *in vivo*

**Figure 8:**
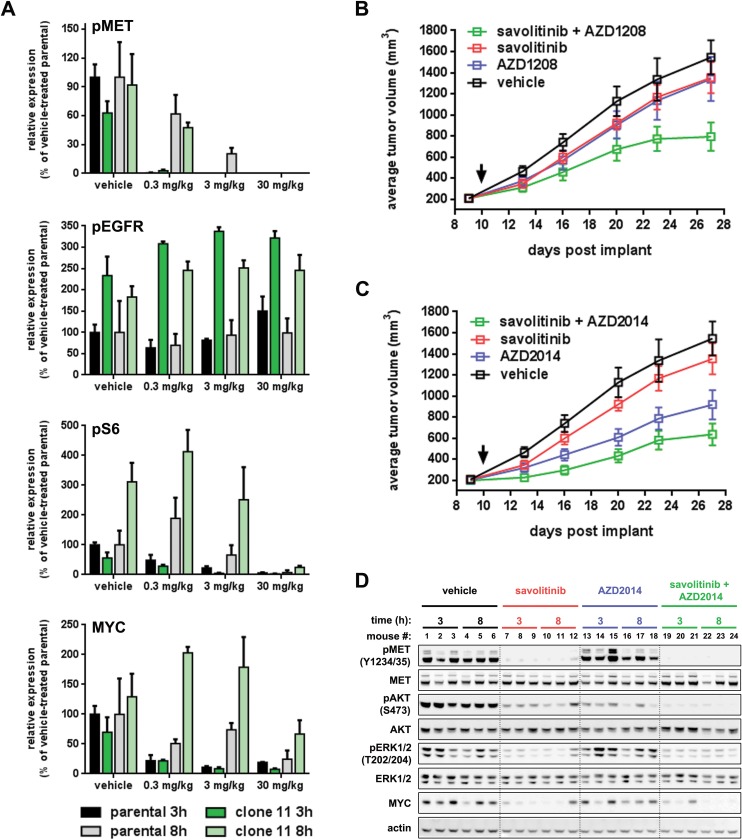
Inhibition of PIM or mTOR kinases reverses savolitinib resistance *in vivo* **A.**, densitometry quantitation of immunoblot data assessing pharmacodynamic changes in parental H1993 tumors (black and grey bars) and clone 11 tumors (light and dark green) following treatment with the indicated doses of savolitinib for three or eight hours. Raw immunoblot data can be found in Supplementary Figure 9B, 9C. **B.**, plot of tumor growth over time for the H1993 clone 11 tumor model. Tumor-bearing mice were randomized into groups (*n* = 10/group) and dosed by oral gavage as follows: 10 mL/kg vehicle twice daily, 30 mg/kg savolitinib once daily, 30 mg/kg AZD1208 twice daily or the combination of savolitinib and AZD1208. Black arrow indicates the start of dosing. Tumors were measured by caliper twice weekly. Data shown are the arithmetic mean of each treatment group ± s.e.m. **C.**, plot of tumor growth over time for H1993 clone 11 tumors treated with AZD2014, as a single-agent or in combination with savolitinib, from the same efficacy study shown in panel B (the vehicle and single-agent savolitinib groups are exactly the same mice as shown in B). AZD2014 was administered orally at 20 mg/kg twice daily for two days followed by a five-day drug holiday before start of the next treatment cycle. Data shown were analyzed as described in B. **D.**, pharmacodynamic analysis of tumor lysates from the H1993 clone 11 study shown in panel C. Tumors from three independent mice were analyzed from each treatment group three and eight hours after dosing.

It is well-established that tumor cell signaling and behavior are context-dependent and gene expression, signaling pathway utilization, growth kinetics and sensitivity/resistance to drugs can vary dramatically between cells grown on plastic *versus* grown *in vivo*. Thus, we first determined whether savolitinib resistance is maintained *in vivo* by implanting H1993 clone 11 cells sub-cutaneously into nude mice and treating tumor-bearing mice with a range of savolitinib doses. Indeed, H1993 clone 11 tumors are insensitive to savolitinib over a 100-fold range of doses, including the clinically-relevant dose of 30 mg/kg (Supplementary Figure S9A). Next, we assessed the expression and modulation of key pharmacodynamic biomarkers in clone 11 tumor lysates at three and eight hours following a final dose with either 0.3, 3 or 30 mg/kg savolitinib; for comparison, lysates from parental H1993 tumors matched for both savolitinib dose and time after adminstration were also analyzed. At three hours post-administration, MET phosphorylation is almost completely inhibited at all doses in both the parental H1993 and clone 11 tumors. MET phosphorylation begins to return to baseline levels by eight hours, with the degree and duration of MET inhibition correlating with dose and MET phosphorylation being fully inhibited for at least eight hours at the 30 mg/kg dose in both tumor models (Figure 8A, top; Supplementary Figure S9B, C).Thus, savolitinib inhibits MET phosphorylation to a similar extent in both the H1993 parental and clone 11 tumor models *in vivo*, consistent with our *in vitro* results.

We also quantified levels of phospho-EGFR (pY1068; pEGFR), phospho-ribosomal protein S6 (pS235/236; pS6) and MYC proteins. Interestingly, the relative levels and modulation of all three proteins in both tumor models are strikingly consistent with our *in vitro* results. pEGFR expression is elevated in clone 11 tumors relative to parental levels and is essentially unchanged by savolitinib treatment at all three doses (Figure 8A, second panel from top; Supplementary Figure S9B, C); total EGFR protein levels are also elevated in clone 11 tumors relative to parental tumors (Supplementary Figure S9B, C), as we observed *in vitro* (Figure 5D). S6 phosphorylation was dose-dependently inhibited to a similar extent in both parental and clone 11 tumors at three hours post-dose, but was elevated two to three-fold above parental levels in all but the 30 mg/kg treatment group (Figure 8A, third panel from top; Supplementary Figure S9B, C). Lastly, we examined MYC expression in both tumor models. At three hours post-dose MYC levels are suppressed to a similar extent in both tumor models, and the degree of MYC suppression correlates with savolitinib dose. However, whereas MYC protein levels return to approximately 50% of baseline levels in parental H1993 tumors eight hours after dosing with 0.3 or 3 mg/kg savolitinib, MYC levels rebound much higher in clone 11 tumors at the same doses, surpassing clone 11 baseline levels and reaching 2-3 fold the levels in parental H1993 tumors (Figure 8A, bottom panel; Supplementary Figure S9B, C). Furthermore, at the highest savolitinib dose tested (30 mg/kg), where both pMET and MYC remain strongly suppressed at eight hours in parental H1993 tumors, MYC expression in the clone 11 model returns to approximately 50-60% of baseline despite complete inhibition of pMET, recapitulating our *in vitro* finding that MYC expression becomes uncoupled from MET activation in the context of savolitinib-resistance. Taken together, these results demonstrate that H1993 clone 11 tumors maintain savolitinib resistance *in vivo*, and that patterns of signaling and savolitinib-induced pharmacodynamic changes observed *in vitro* are also observed *in vivo*.

### Inhibition of PIM or mTOR kinases restores savolitinib sensitivity *in vivo*

Having established that the H1993 clone 11 model retains its core characteristics *in vivo*, we next tested whether our *in vitro* findings could be extended *in vivo* by testing the effects of AZD1208 and AZD2014 treatment, alone or in combination with savolitinib, on the growth of H1993 clone 11 tumors. Nine days after sub-cutaneous implantation of H1993 clone 11 cells, tumor-bearing nude mice were randomized into treatment groups of ten mice per group. Treatment began the following day with each drug — AZD1208 (30 mg/kg, BID), AZD2014 (20 mg/kg, BID, 2on/5off) and savolitinib (30 mg/kg, QD) — given alone or in combination with savolitinib. After 17 days of dosing, treatment with the pan-PIM inhibitor AZD1208 alone or savolitinib alone resulted in statistically-insignificant, tumor growth inhibition (TGI) of ~12% ± 9.6% s.e.m. relative to the vehicle-treated group (Figure 8B). However, combination of savolitinib and AZD1208 resulted in a TGI of 51% ± 8.7% s.e.m. relative to the vehicle group, similar to the maximum TGI observed with savolitinib treatment alone (~59% ± 6.1% s.e.m.) in the parental H1993 tumor model (Figure 2A). These data suggest that in the context of abrogated MET signaling, PIM kinase inhibition is sufficient to restore sensitivity to savolitinib in a xenograft model of acquired savolitinib resistance. Lastly, we examined the effect of combining the dual mTORC1/2 inhibitor AZD2014 with savolitinib on H1993 clone 11 tumor growth. *In vitro*, clone 11 cells show no response to savolitinib or AZD2014 as single agents but the combination synergized to reduce cell viability (Figure 6B). In contrast, *in vivo* we found that AZD2014 alone reduced H1993 clone 11 tumor growth by ~41% ± 8.9% s.e.m. (Figure 8C) which correlated with suppression of AKT activation (Figure 8D). Combination of AZD2014 with savolitinib generated a small supra-additive effect, increasing the TGI to ~59% ± 6.6% s.e.m. and resulting in an even greater degree of pAKT suppression.

## DISCUSSION

The present study explored the therapeutic potential of savolitinib — a potent, selective small-molecule MET inhibitor — in NSCLC. We demonstrate that savolitinib inhibits MET i*n vitro* and *in vivo* and reduces viability of MET-dependent models harboring *MET* CN gain. Savolitinib efficacy correlates with downstream inhibition of the PI3K/AKT and MAPK pathways and MYC downregulation. Additionally, we generated savolitinib resistance in NSCLC models and found savolitinib resistance is stable over time and arises without activating *MET* mutations. We also independently confirm the recent novel finding that uncoupling of MYC expression from MET signaling is required for TKI resistance. Lastly, we show that constitutive mTOR pathway activation can confer savolitinib resistance in multiple clones, whereas acquired EGFR or PIM dependency are clone-specific mechanisms.

Tumor cells harboring *MET* copy number gains are often rendered dependent on MET signaling for survival and proliferation, and are therefore sensitive to MET inhibition [20]. Our analysis of the nearly one thousand cell lines present in the Sanger Cell Line Panel confirms this phenomenon, as those cell lines most sensitive to savolitinib are also those with *MET* CN gains, although a small number of outlier cell lines are evident. First, we note two cell lines with high *MET* CN lacking savolitinib sensitivity (OE33 and CS-1) that potentially represent models of intrinsic savolitinib resistance. While almost nothing is known about the genetics of CS-1 cells, a more detailed analysis of the mutational landscape of OE33 cells revealed, among other variations, a striking *HER2* (*ERBB2*) amplification of approximately 14 copies [43]. It is tempting to speculate that aberrant HER2 activity acts as a bypass mechanism to render OE33 cells innately resistant to savolitinib [44–45]. Validation of this hypothesis would suggest that hyperactive EGFR family signaling is a common mechanism shared by intrinsic and acquired savolitinib resistance.

Conversely, we found two cell lines with low or no *MET* CN gain but savolitinib GI_50_ values below 1.0 μM (EW-11 and MV411). The precise mechanism(s) conferring such savolitinib sensitivity are not readily apparent for these cell lines; however, one study looking at determinants of sensitivity to another MET inhibitor, PHA-665752, found that while *MET* amplification was a strong predictor of MET inhibitor sensitivity, high levels of MET phosphorylation in the absence of *MET* amplification can also predict ‘intermediate’ sensitivity to PHA-665752 [46]. In sum, our analysis of savolitinib sensitivity across a large and diverse panel of cell lines confirms previous findings and demonstrates that while high *MET* CN gain is a strong predictor of savolitinib sensitivity, MET CN-independent factors can influence savolitinib sensitivity. Inclusion of such criteria will be critical to the success of savolitinib clinical trial design and patient stratification strategies in the future.

Previous studies of acquired resistance to MET inhibitors have shown that activating mutations in *MET* or additional *MET* CN gains can drive resistance [47–49]. However, MET phosphorylation remains sensitive to inhibition by savolitinib in our resistant cell lines, arguing against *MET* mutation as a resistance mechanism. Exome sequencing of 45 genes, including *MET*, confirmed the absence of *MET* mutations but did reveal *MET* CN gains in some resistant clones; however, the biological significance of such *MET* gains is unclear given that MET is equally inhibited in all resistant clones irrespective of *MET* CN.

TKI resistance can arise through aberrant activation of the PI3K/AKT/mTOR axis in several cancer types, including NSCLC, often through *PTEN* deletion [50] or activating *PIK3CA* mutations [51]; however, such mechanisms were elucidated primarily in the context of EGFR inhibitors. Here, we demonstrate that deregulated PI3K/AKT/mTOR activity also confers MET inhibitor resistance in NSCLC, but without *PTEN* loss or *PIK3CA* mutation, suggesting that an uncommon or novel mTOR activation mechanism may underlie savolitinib resistance.

Additionally, we observe MYC upregulation in all resistant H1993 clones and in a resistant EBC-1 population, suggesting that MYC may be a critical node upon which more diverse mechanisms converge. Indeed, we demonstrate that constitutive, elevated MYC expression actively promotes resistance to savolitinib in H1993 and EBC-1 cells. Interestingly, during preparation of this manuscript, Shen and colleagues reported that MYC can promote acquired resistance to another MET inhibitor, SGX-523 [52]. Despite the fact that clinical development of SGX-523 was discontinued due to unexpected tolerability concerns, their work supports our findings regarding acquired savolitinib resistance. Taken together, our data and those of Shen et al. suggest that inhibiting MYC activity may be a strategy to prevent or reverse potential savolitinib resistance in the clinic. Importantly, although MYC has long been considered an ‘undruggable’ protein, recent development of a small-molecule MYC inhibitor tool compound may hold promise for a future MYC-based therapy [53]. Alternatively, indirect abrogation of MYC activity by inhibiting members of the BET bromodomain-containing family of proteins [54–55] may offer a means to reverse savolitinib resistance.

A switch to MET signaling can drive acquired resistance to EGFR inhibitors, such as erlotinib [56] and gefitinib [57], in NSCLC, and EGFR signaling can drive MET inhibitor resistance [25–26], illustrating the reciprocal nature of signaling between these RTKs. Indeed, we observe a clone-specific reversible switch to EGFR dependence demonstrated by synthetic lethality of combined MET/EGFR inhibition; furthermore, our data suggest that EGFR and/or HER2 signal to MET in resistant, but not parental, H1993 cells while MET appears to partially activate EGFR in a clone-specific manner. Importantly, the discordance we observe between EGFR expression/activation levels and EGFR inhibitor sensitivity illustrates a key concept in drug resistance studies - gene expression and activity per se are not infallible predictors of therapeutic responses. Instead, *functionally* assessing the relationships between gene products and drug resistance can reveal actionable, perhaps unexpected, vulnerabilities in drug-resistant cells regardless of gene expression, copy number, mutation or activation status [58].

Lastly, we demonstrate a role for PIM kinases in savolitinib resistance. Importantly, and to the best of our knowledge, our study and that of An et al [42] are the first ever to report a role for PIM signaling in the context of acquired MET inhibitor resistance. Using two structurally-distinct small-molecule pan-PIM inhibitors, we show that blocking PIM activity concurrent with MET inhibition resensitizes savolitinib-resistant cells *in vitro.* Interestingly, when we assessed the *in vivo* efficacy of combining the PIM inhibitor with savolitinib, in savolitinib resistant H1993 clone 11 tumors we observed similar anti-tumor activity as seen with savolitinib alone in H1993 parental tumors. This demonstrates that inhibition of PIM concurrent with MET inhibition resensitizes savolitinib-resistant tumors *in vivo* as well.

Intriguingly, clone 11 is the only clone sensitive to EGFR or PIM inhibition in the presence of savolitinib, leading us to hypothesize that that this particular clone may utilize a novel signaling pathway involving EGFR and PIM to circumvent MET inhibition. In support of this notion, Siu and colleagues previously demonstrated in prostate cancer models that PIM-1 inhibition upregulates MIG6 [59], a negative regulator of EGFR signaling [60], thereby inhibiting EGFR/MAPK activation. In line with these findings and our hypothesis, clone 11 cells treated with savolitinib alone display increased pEGFR levels, consistent with a switch to EGFR signaling when MET is inhibited. Treatment with a PIM inhibitor is sufficient to block this increase in EGFR activation and results in concomitant downregulation of MYC and pS6 levels. Further work will be needed to characterize the precise nature of how PIM and EGFR signaling interact to mediate savolitinib resistance.

The treatment of NSCLC with evermore potent and selective therapies is a boon to patients and a testament to our increasing knowledge of oncogenesis. Our study demonstrates savolitinib efficacy in preclinical NSCLC models representing adenocarcinoma, squamous cell carcinoma and metastatic disease. We uncover acquired resistance mechanisms in common between clones as well as clone-specific mechanisms, including identification of PIM as a novel mediator of resistance, laying the groundwork for combination strategies to combat savolitinib resistance that may arise in the clinic. Importantly, we illustrate the heterogeneity of potential resistance mechanisms within a single population of tumor cells, underscoring the need for careful molecular characterization of tumors from relapsed patients.

## MATERIALS AND METHODS

### Tissue culture and savolitinib-resistant cell line generation

NCI-H1993 (H1993) and NCI-H2009 (H2009) were purchased from ATCC (Rockville, MD). EBC-1 cells were purchased from the Japanese Collection of Research Bioresources (JHSF). All lines were authenticated by STR analysis (IDEXX BioResearch, Columbia, MO) and used within six months of receipt. Cells were cultured in RPMI-1640 (Sigma-Aldrich) containing 10% FBS at 37°C/5% CO_2_ in a humidified atmosphere. Savolitinib-resistant cells were generated by sequentially increasing the savolitinib concentration during culture until cells grew in the presence of 2.0 μM compound.

### *MET* copy number analysis in NSCLC

Gene copy number (CN) data for LUAD and LUSC cohorts (TCGA) were used to estimate *MET* CN frequency in NSCLC. *MET* CN gain was classified using both focality and amplitude, and samples were subsequently divided into either low-level (CN >2 but ≤3) or high-level (CN>3).

### Sanger cell line screening

Cell line screening was performed as previously described [21].

### Immunoblot analysis

Preparation of protein lysates and immunoblot analyses were performed as described previously [19]. Please refer to Supplementary Table 2 for a list of antibodies.

### Small-molecule sensitivity assays

Drug treatment, cell viability measurements, curve fitting and IC_50_ calculations were performed as described in [19]. Please refer to Supplementary Table 1 for a full list of compounds used in this study.

### Cell viability assays

All cell viability measurements were performed as described in [19] using Cell Titer Glo assay (Promega, catalog# G7570).

### *In vivo* efficacy and pharmacodynamic studies

For H1993 and EBC-1 xenografts: 6-7 week-old female Balb/c nu/nu mice (Shanghai Laboratory Animal Co. Ltd.; certification#: 2007000506076) were maintained in a controlled, specific pathogen-free environment at 20-25°C, 40-70% humidity and a photoperiod of 12 hours light-to-dark. Each mouse was injected subcutaneously in the right lateral flank with 5×10^6^ cells suspended in 0.2 mL 1:1 matrigel and randomized based on tumor volume; dosing began when tumors reached 120-275 mm^3^. For HLXF-036LN PDX studies (lymph-node metastasis from a 61 year-old male with lung adenocarcinoma - stage IIA, grade 3, mixed acinar, papillary and micropapillary - procured from Maine Medical Center, Portland, ME), tumor fragments were passaged into NSG mice (The Jackson Laboratory) and allowed to grow to an average size of 250.0 mm^3^ and then randomized. Savolitinib was formulated in acidic CMC-Na 0.5% (pH = 2.1) and dosed orally once daily at the indicated concentrations for all studies. For pharmacodynamic analysis, tumors were lysed in ten volumes of lysis buffer containing protease and phosphatase inhibitors and dounce homogenized. Samples were centrifuged at 20,000*g* for 10′ at 4°C and the supernatants transferred to a new microfuge tube three times. Lysates were quantified by BCA assay and subjected to immunoblotting. All animal studies were performed according to AstraZeneca Institutional Animal Care and Use Committee guidelines.

### Phospho-protein array

Cells were incubated overnight without savolitinib, treated with 2 μM savolitinib for four hours and then lysed and subjected to the PathScan^®^ RTK Signaling Antibody Array exactly as instructed by the manufacturer (catalog# 7982S, CST, Inc, Bedford, MA). Chemiluminescence was captured on an ImageQuant LAS 4000 instrument (Fuji). Densitometry analysis was performed using the ImageQuant TL Array software, V8.1 (GE Healthcare).

### MYC knockdown

Ambion Silencer Select siRNAs (human *MYC:* s9130 and s9129; non-targeting control #4390843) were reverse-transfected at 5 nM final concentration with Lipofectamine RNAimax (Life Technologies). *MYC* transcript levels were measured 72 hours later by qRT-PCR (normalized to *GAPDH*) - Taqman probes: *GAPDH* #1404051, *MYC* Hs00153408_m1 (Life Technologies). For viability experiments, cells were transfected with siRNA, exposed to DMSO or savolitinib 24 hours later, and viability was assessed after three days.

### MYC overexpression

H1993 RFP- and MYC-overexpressing cell lines were generated by lentiviral transduction of the respective tet-inducible pTRIPZ vector (ThermoScientific), selection with 0.5 μg/ml puromycin (Sigma-Aldrich) three days post-infection followed by culture in RPMI-1640 medium containing tetracycline-free serum and 0.5 μg/ml puromycin. For proliferation assays, 2,000 cells/well were plated in 96-well plates +/− 1 μg/ml doxycycline and treated with DMSO or savolitinib 24 hours later; viability was measured after five days. For immunoblot analyses, 5×10^5^ cells were plated in 6-well plates +/− 1 μg/ml doxycycline and treated with DMSO or 100 nM savolitinib 24 hours later. After an additional 24 hours, cells were collected and protein expression was measured by immunoblot.

## SUPPLEMENTARY MATERIAL FIGURES AND TABLES


