# Supplementary material for: Acquired savolitinib resistance in non-small cell lung cancer arises via multiple mechanisms that converge on MET-independent mTOR and MYC activation
Source: Oncotarget. 2016 Jul 26;7(36):57651–70. doi: 10.18632/oncotarget.10859 (PMC5295379; doi:10.18632/oncotarget.10859)
Supplement: Supplementary file 1 [file oncotarget-07-57651-s001.pdf]

Acquired savolitinib resistance in non-small cell lung cancer arises via multiple mechanisms that converge on MET-independent mTOR and MYC activation

Supplementary Material

- **SUPPLEMENTARY FIGURES S1-S9**
- **SUPPLEMENTARY MATERIALS AND METHODS**
- **SUPPLEMENTARY TABLES S1 and S2**
- **SUPPLEMENTARY REFERENCES**

Supplementary Figure S1

Figure S1

A

Cell line	GI_{50} (µM) (Sanger)	GI_{50} (µM) (Hutchison Medi Pharma)	*MET* status	*MET* copy number (Sanger, COSMIC)	cancer type
EBC-1	0.00004	0.002	amplified	14	lung squamous cell carcinoma
ESO51	0.00040	N.D.	amplified	12	esophageal adenocarcinoma
SNU5	0.00140	0.003	amplified	14	stomach
MKN45	0.00620	0.004	amplified	10	stomach
H1993	0.08360	0.010	amplified	14	lung adenocarcinoma
Hs746T	0.31410	0.005	amplified	11	gastric adenocarcinoma

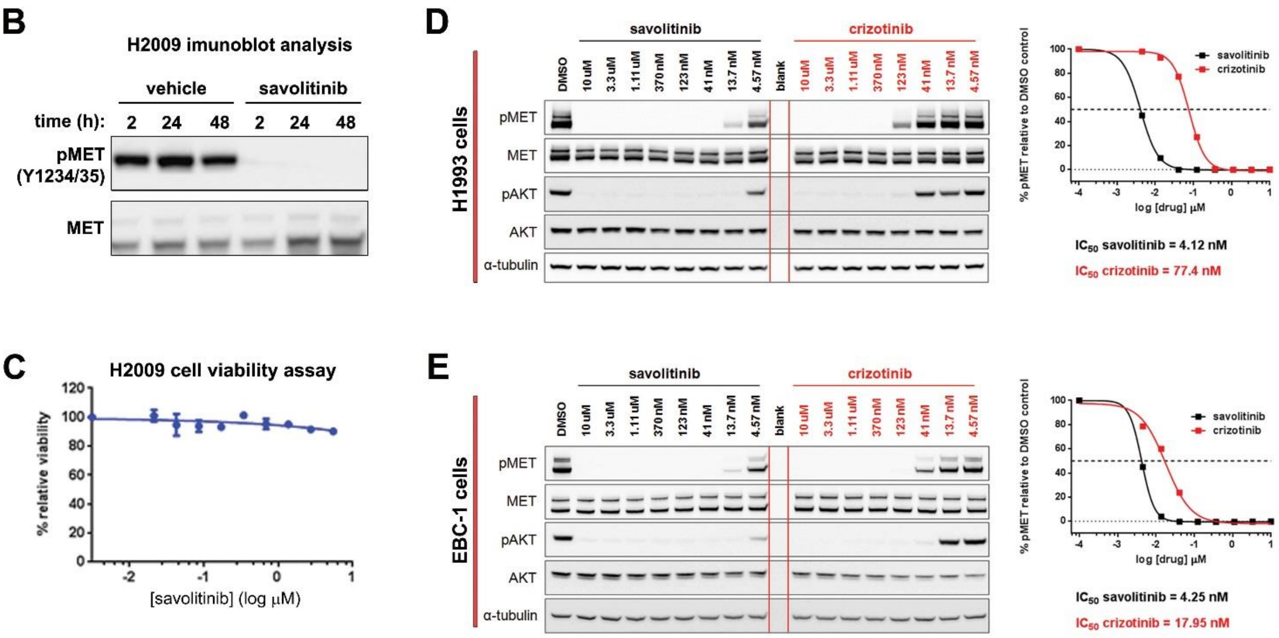

Supplementary Figure S1. A, table summarizing savolitinib sensitivity, *MET* CN status and cancer subtype data for the six most sensitive cell lines. *MET* status and copy number data were obtained from the COSMIC database (http://cancer.sanger.ac.uk/cosmic). N.D., not determined. B, western blot analysis of total and activated (phosphorylated) MET levels in the H2009 NSCLC line (intrinsically savolitinib resistant) treated with vehicle or 100 nmol/L savolitinib for the indicated times. C, viability of H2009 cells treated with a dose range of savolitinib for four days. Data are normalized to a vehicle-treated control and represent the mean +/- S.D. of duplicate experiments. D, immunoblot analysis (left) and densitometry quantitation (right) of phospho-MET protein levels in H1993 cells treated with the indicated concentrations of savolitinib or crizotinib for two hours. pMET refers to MET phosphorylated at Y1234/1235. Phosphorylation of the downstream kinase AKT on S473 (pAKT) is also shown. α-tubulin served as a loading control. For quantitation of pMET inhibiton, total MET and pMET levels were first normalized to α-tubulin. pMET/total MET ratios were then calculated for each treatment. The pMET/total MET ratio for DMSO treatment was set equal to 1 and all other ratios were expressed as a percentage of the DMSO control. E, the experiment shown in D was repeated exactly as described but in EBC-1 cells.

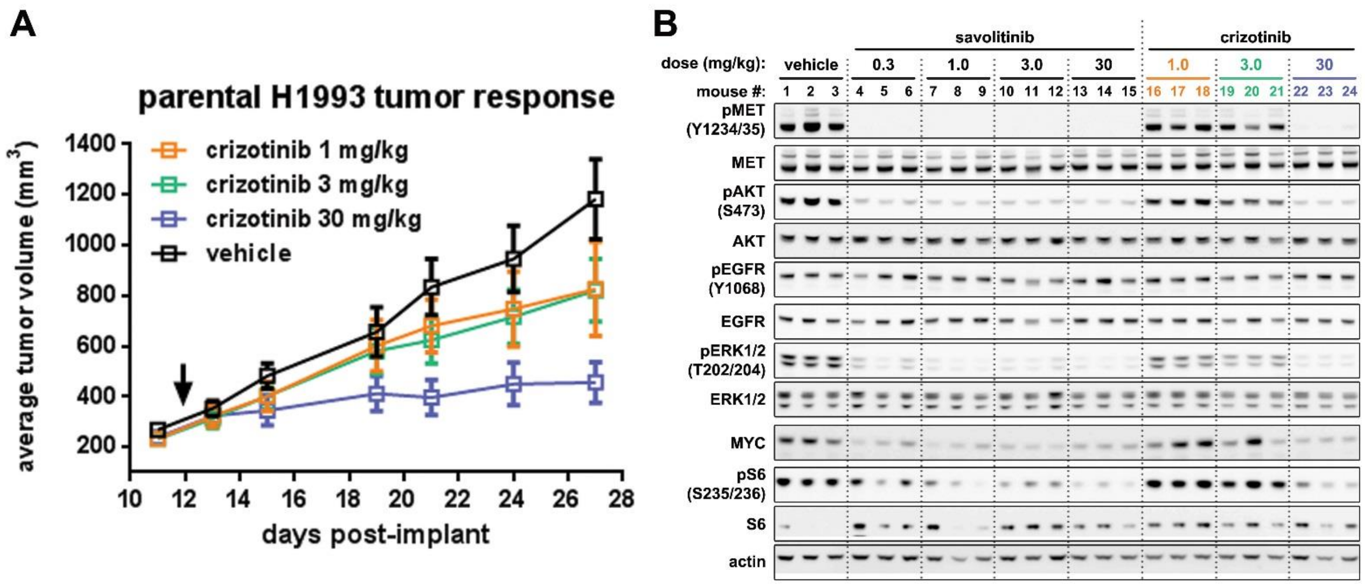

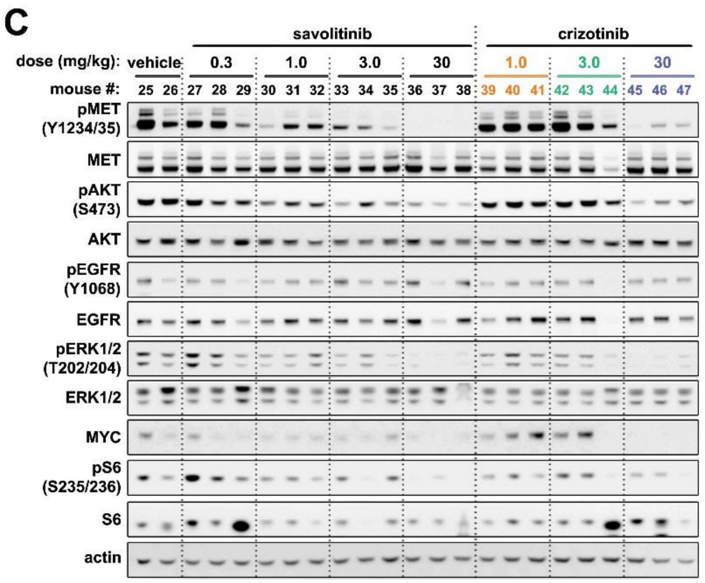

Supplementary Figure S2. A, plot of tumor growth over time for the parental H1993 tumor model. Tumor-bearing mice were randomized into groups (n=10/group) and treated with vehicle or the indicated doses of crizotinib once daily by oral gavage. Black arrow indicates the start of dosing. Tumors were measured by caliper twice weekly. Data shown are the arithmetic mean of each treatment group ± s.e.m. B-C, pharmacodynamic analysis of lysates from parental H1993 tumors three hours (B) and eight hours (C) after treatment with vehicle or the indicated doses of savolitinib or crizotinib. Tumors from three independent mice were analyzed for each treatment except for the vehicle-treated group at eight hours (n=2).

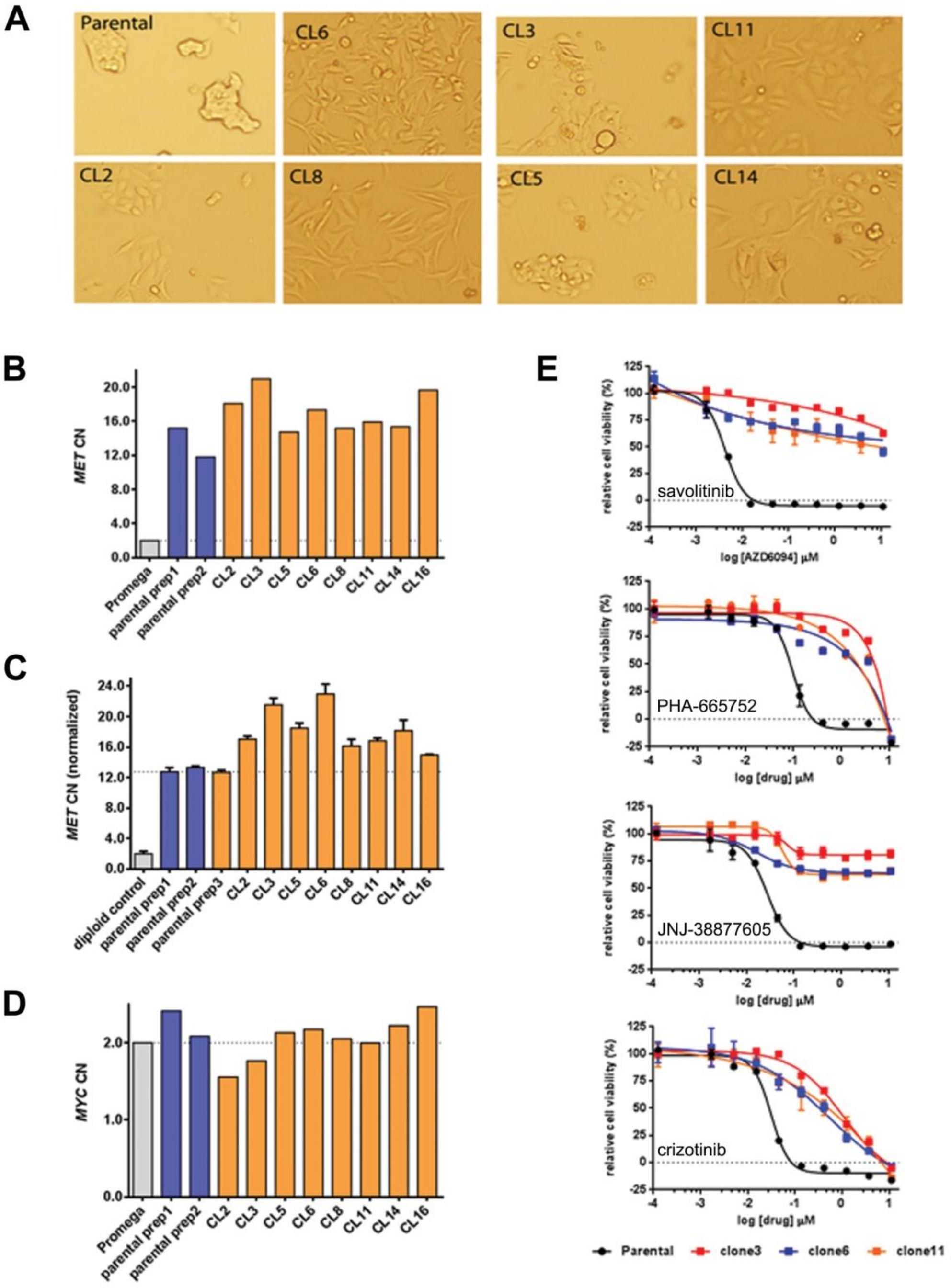

Supplementary Figure S3. A, brightfield microscopy images of parental H1993 cells and seven savolitinib-resistant clones showing cell morphological differences. B, next-generation sequencing analysis of *MET* copy number in parental H1993 cells and eight savolitinib-resistant clones. A diploid control (Promega) is shown for reference. C, verification of *MET* CN in parental and savolitinib-resistant H1993 cells by quantitative PCR. Diploid control is gDNA from the BJ fibroblast cell line. D, next-generation sequencing analysis of *MYC* copy number in parental H1993 cells and eight savolitinib-resistant clones. A diploid control (Promega) is shown for reference. E, viability of parental H1993 cells and savolitinib-resistant clones 3, 6 and 11 treated with a dose range of the MET inhibitors savolitinib, PHA-665752, JNJ-38877605 and crizotinib for five days. Data are normalized to vehicle-treated controls.

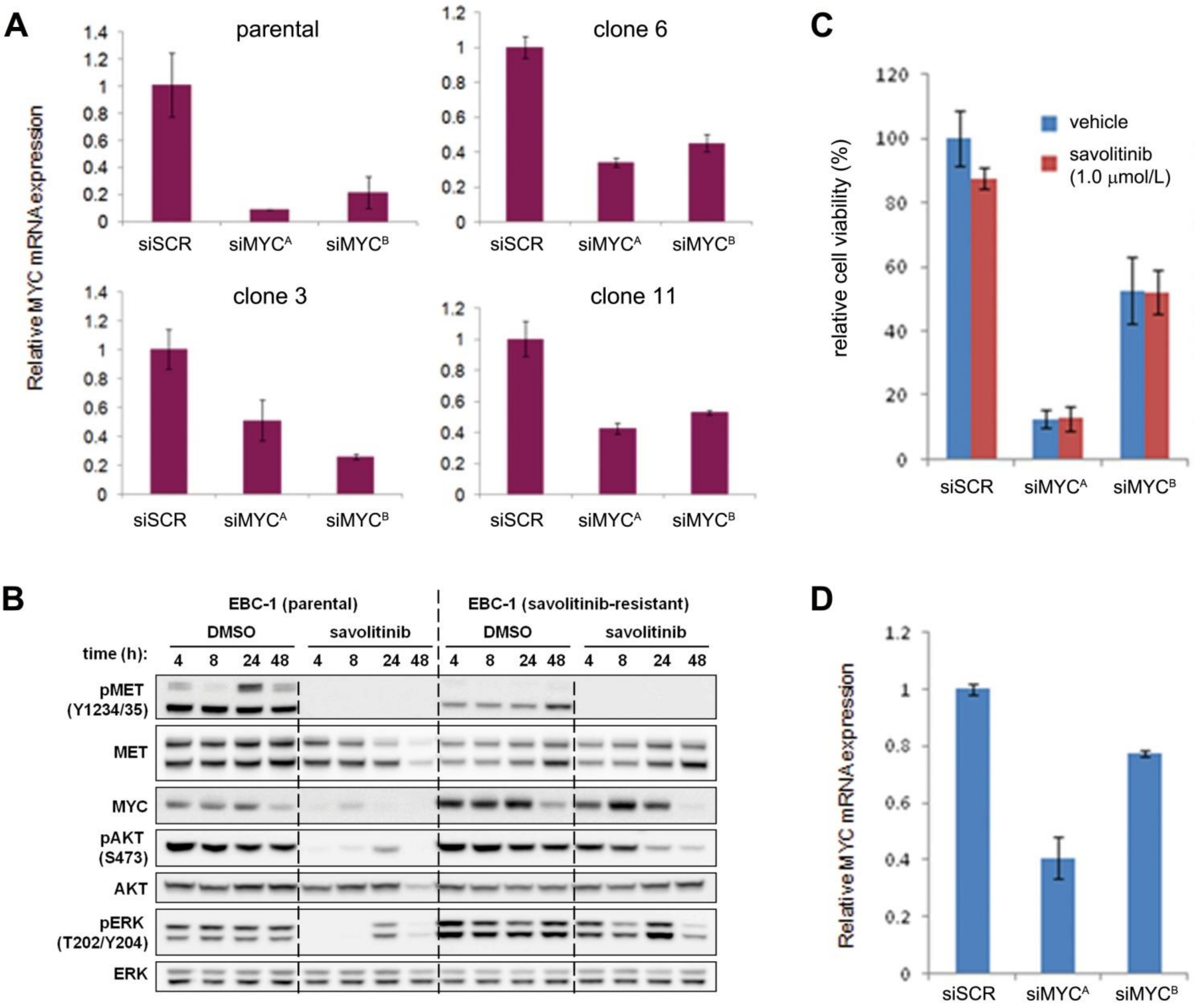

Supplementary Figure S4. A, MYC mRNA expression in parental and savolitinib-resistant H1993 cells as measured by quantitative reverse-transcriptase PCR following transfection with non-targeting (siSCR) or MYC-targeting (MYC^A, MYC^B) siRNAs. B, immunoblot characterization of an EBC-1 cell population with acquired savolitinib resistance. Cells were treated with vehicle (DMSO) or 100 nmol/L savolitinib for the indicated times. Total ERK serves as a loading control. C, cell viability measurements following MYC knockdown and savolitinib treatment in parental and savolitinib-resistant EBC-1 cells. Parental EBC-1 cells and a savolitinib-resistant population were transfected with a negative control siRNA (siSCR) or each of two MYC-targeting siRNAs ($siMYC^A$, $siMYC^B$) and treated with vehicle or 100 nmol/L savolitinib. Cell viability was assessed three days post-savolitinib treatment. Data shown are the mean +/- S.D. of three replicates. D, relative MYC mRNA expression in parental and savolitinib-resistant EBC-1 cells by quantitative reverse-transcriptase PCR following transfection with non-targeting (siSCR) or MYC-targeting ($siMYC^A$, $siMYC^B$) siRNAs.

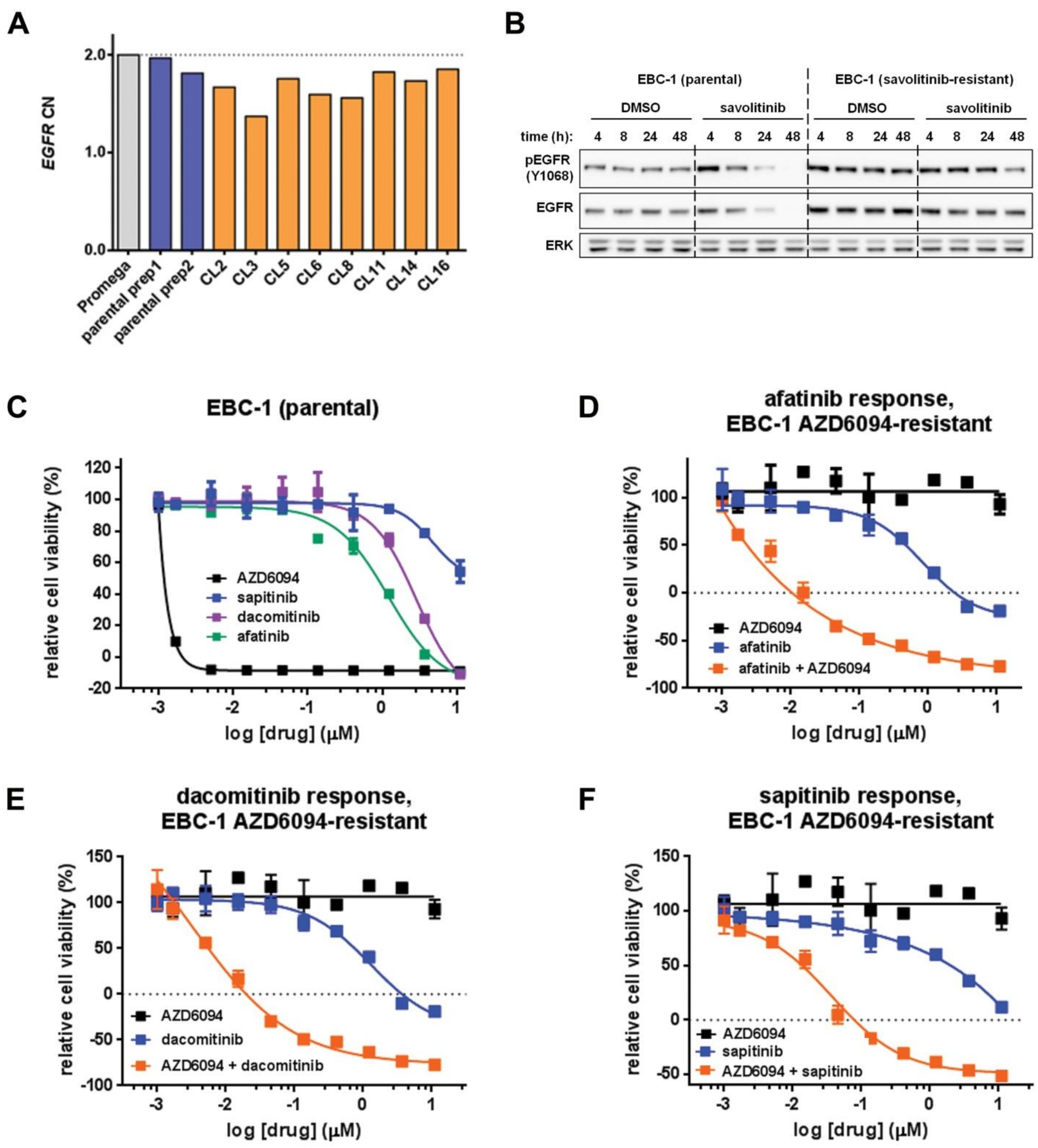

Supplementary Figure S5. **A**, next-generation sequencing analysis of *EGFR* copy number in parental H1993 cells and eight savolitinib-resistant clones. Diploid control DNA (Promega) is shown for reference. **B,** immunoblot analysis of parental and savolitinib-resistant EBC-1 cells treated with 100.0 nmol/L savolitinib or 0.1% DMSO (vehicle control) for the indicated time points. Total ERK serves as a loading control. **C-F,** 5-day cell viability assays of cells treated with EGFR inhibitors. Dose response data for parental EBC-1 cells (C) treated with savolitinib or each of three EGFR family inhibitors. **D-F,** dose response data for savolitinib, an EGFR inhibitor or the combination in savolitinib-resistant EBC-1 cells. Combination of savolitinib and EGFR inhibitors synergistically reduces cell viabilty of savolitinib-resistant EBC-1 cells. For combination treatments, savolitinib concentration is held constant at 100 nmol/L while EGFR inhibitor concentrations vary.

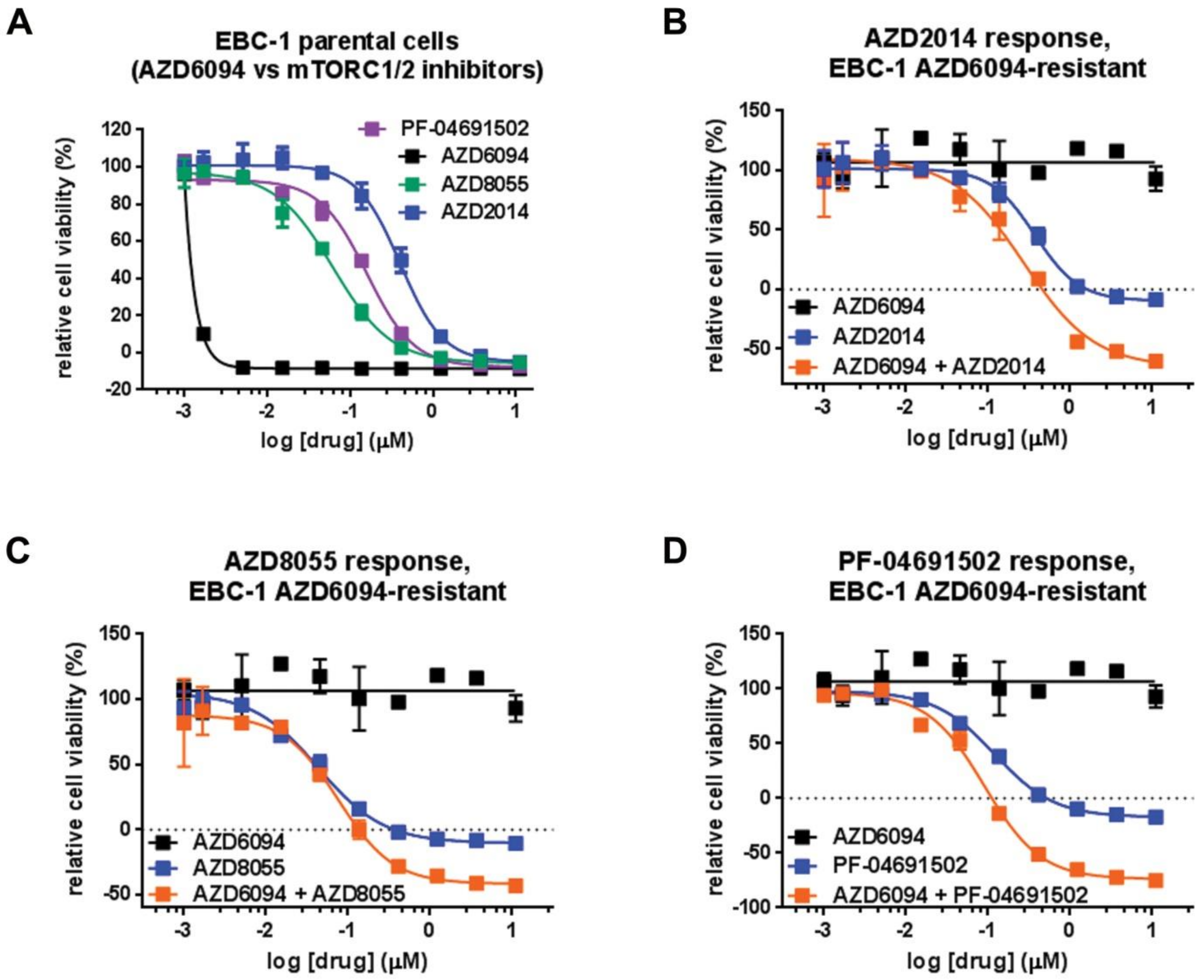

Supplementary Figure S6: 5-day cell viability assays of cells treated with dual mTORC1/2 inhibitors. **A,** dose response data for parental EBC-1 cells treated with savolitinib or each of three dual mTORC1/2 inhibitors. **B-D,** dose response data for savolitinib, a dual mTORC1/2 inhibitor or the combination in savolitinib-resistant EBC-1 cells. Combination of savolitinib and a dual mTORC1/2 inhibitor synergistically reduces cell viabilty of savolitinib-resistant EBC-1 cells at higher concentrations. For combination treatments, savolitinib concentration is held constant at 100 nmol/L while mTORC1/2 inhibitor concentrations vary.

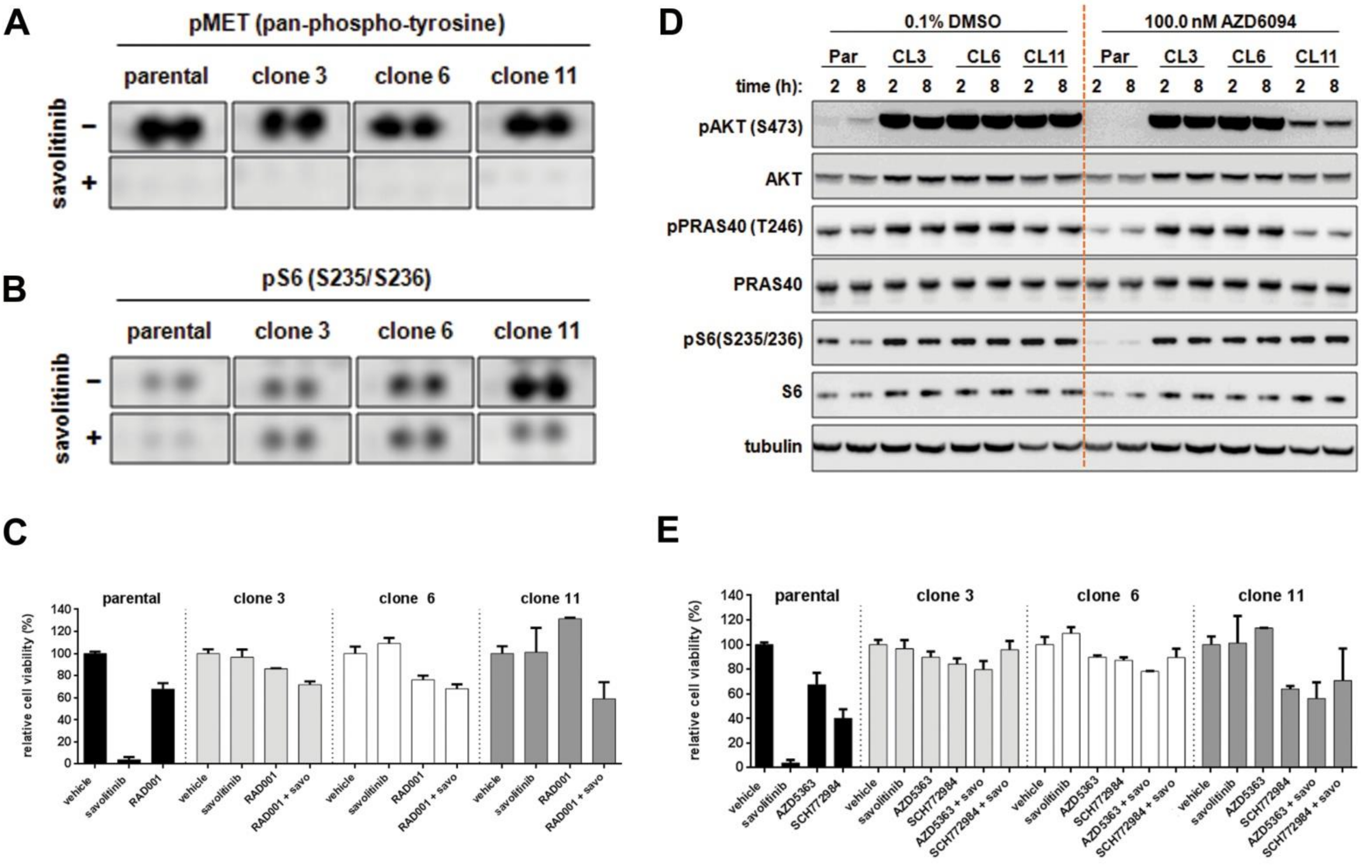

Supplementary Figure S7. A-B, phospho-protein array images for MET and S6. Film scans of array results for pMET (A) and pS6 (Ser235/Ser236) (B). Capture antibodies are present in duplicate on each array. C, five-day cell viability assay of parental H1993 cells and savolitinib-resistant H1993 clones treated with 412 nmol/L of the mTORC1-specific inhibitor RAD001 in the presence or absence of 100 nmol/L savolitinib. D, western blot analyis of parental H1993 cells (Par) or savolitinib-resistant clones (CL3, 6 and 11) treated with vehicle (0.1% DMSO) or 100 nmol/L savolitinib (AZD6094) for the indicated times. α-tubulin serves as a loading control. E, five-day cell viability assay of parental H1993 cells treated with single-agent savolitinib (100 nmol/L) or the indicated inhibitors at 412 nmol/L. Savolitinib-resistant clones 3, 6 and 11 were treated with 412 nmol/L of each inhibitor alone or in combination with 100 nmol/L savolitinib.

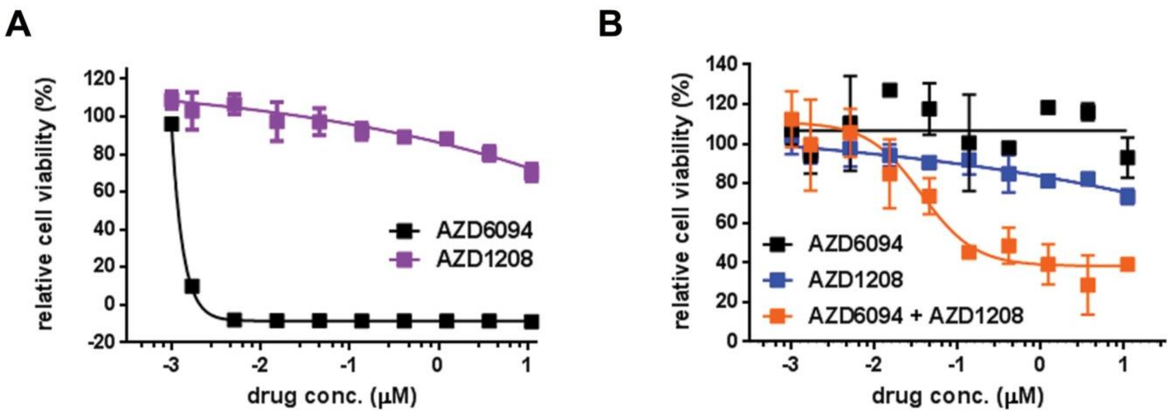

Supplementary Figure S8: 5-day cell viability assays of cells treated with the pan-PIM inhibitor AZD1208. **A,** dose response data for parental EBC-1 cells treated with savolitinib or AZD1208. **B,** dose response data for savolitinib, AZD1208 or the combination in savolitinib-resistant EBC-1 cells. Combination of savolitinib and AZD1208 synergistically reduces cell viabilty of savolitinib-resistant EBC-1 cells. For combination treatments, savolitinib concentration is held constant at 100 nmol/L while AZD1208 concentrations vary.

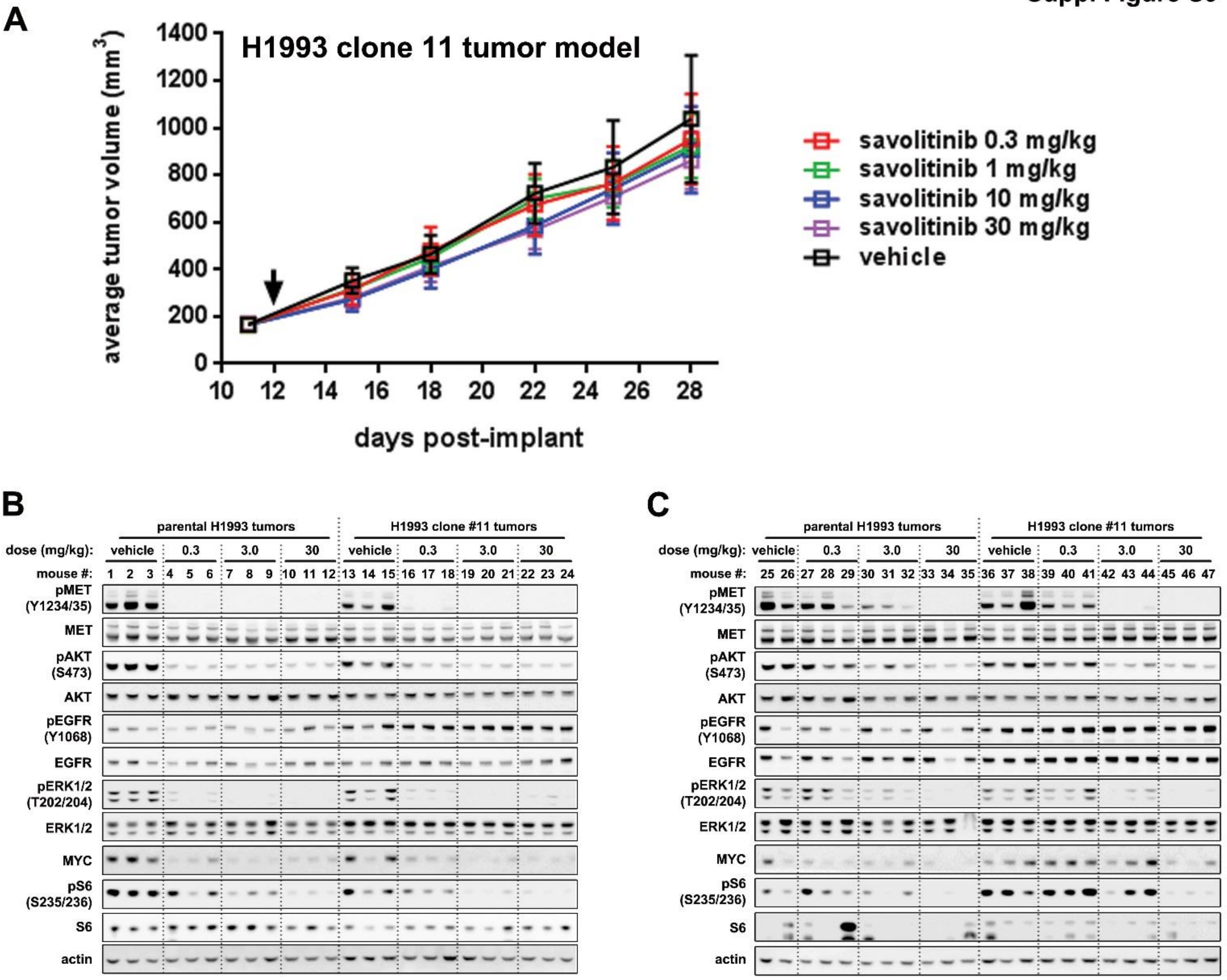

Supplementary Figure S9. A, plot of tumor growth over time for the H1993 clone 11tumor model. Tumor-bearing mice were randomized into groups (n=10/group) and treated with vehicle or the indicated doses of savolitinib once daily by oral gavage. Black arrow indicates the start of dosing. Tumors were measured by caliper twice weekly. Data shown are the arithmetic mean of each treatment group ± s.e.m. B-C, pharmacodynamic analysis of lysates from parental H1993 and clone 11 tumors three hours (B) and eight hours (C) after treatment with vehicle or the indicated doses of savolitinib. Tumors from three independent mice were analyzed for each treatment except for the vehicle-treated group at eight hours where n=2.

Pharmacodynamic analysis of H1993 tumor lysates

Tumors were harvested and lysed in cell lysis buffer containing protease and phosphatase inhibitors and homogenized. Samples were analyzed by ELISA assay according to the manufacturer's protocol (R&D Systems; catalog# DYC358 for total-MET and catalog# DYC2480 for phosphorylated-MET (p-MET). pEGFR/EGFR, pAKT/AKT, pERK/ERK levels were determined by immunoblot and quantified by densitometry.

Quantitative PCR gene copy number analysis

Genomic DNA (gDNA) was prepared using the DNeasy® Blood and Tissue DNA kit (Qiagen, catalog #69504) according to the manufacturer's protocol. Briefly, frozen tumor chunks were weighed on an analytical balance and 20-25 mg of tissue per sample was subjected to the gDNA isolation protocol. gDNA was eluted in 200 µL of elution buffer (provided with kit) and gDNA concentration quantified using a NanoDrop 1000 spectrophotometer (NanoDrop Products). All gDNAs were diluted to 5.0 ng/µL in nuclease-free H_2O. Gene copy number was determined by multiplexed quantitative PCR (qPCR) using a FAM-labeled Taqman probe targeting human *MET* (Hs05018546_cn), *MYC* (Hs02758348_cn) and *EGFR* (Hs02309320_cn). A VIC-labeled probe targeting human *RNAse P1* served as an internal normalization control gene. qPCR was carried out in 384-well format (ABI, part #4309849) sealed with optically clear adhesive film (ABI, part # 4311971), and included the following components per 10 µL reaction:

5 µL - Taqman Gene Expression Master Mix (ABI, part #4369016).

0.5 µL - FAM-labeled gene-specific CN assay probe

0.5 µL - VIC-labeled *RNAse P1* CN assay probe (ABI, part #4401631).

2 µL - nuclease-free H_2O (Ambion, part #AM9906).

2 µL - gDNA template (10 ng total)

Thermocycling conditions on an ABI 7900HT Sequence Detection System run in Standard Mode were as follows:

50°C, 2 min. x 1 cycle

95°C, 10 sec. x 1 cycle

95°C, 15 sec.
60°C, 1 min. } **x 40 cycles**

A standard curve ranging from 80 to 0.3125 ng/well was employed, allowing for *gene-of-interest* and *RNAse P1* ng values to be calculated for each well using the Absolute Quantification (AQ) method. The *gene-of-interest:RNAse P1* ng ratio was calculated for each tumor sample and normalized to that of a diploid fibroblast control sample with a *gene-of-interest:RNAse P1* ratio of 1.0. All ratios were multiplied by two to obtain actual *gene-of-interest* CN values (diploid control contains two copies of each gene).

Sanger cell line compound screening

Cell panel screening is based on previously described methods [1]. All cell lines were sourced from commercial vendors. Cells were grown in RPMI or DMEM/F12 medium supplemented with 5% or 10% FBS and penicillin/streptomycin, and maintained at 37°C in a humidified atmosphere at 5% CO2. Cell lines were propagated in these two media in order to minimize the potential effect of varying the media on sensitivity to therapeutic compounds in our assay, and to facilitate high-throughput screening. To exclude cross-contaminated or synonymous lines, a panel of 92 SNPs was profiled for each cell line (Sequenom, San Diego, CA) and a pair-wise comparison score calculated. In addition, to confirm the identity of each cell line we performed short tandem repeat (STR) analysis (AmpFlSTR Identifiler, Applied Biosystems, Carlsbad, CA) and matched this to an existing STR profile generated by the providing repository.

Compounds were generally stored as 10 mmol/L aliquots at -80°C, and were subjected to a maximum of five freeze-thaw cycles. The range of concentrations selected for each compound was based on in vitro data of concentrations inhibiting relevant kinase activity and cell viability.

Cells were seeded in 384-well microplates at ~15% confluency in medium with 5% FBS and penicillin/streptavidin. The optimal cell number for each cell line was determined to ensure that each was in growth phase at the end of the assay. For adherent cell lines, after overnight incubation cells were treated with five concentrations of each compound (2-fold dilutions series over a 256-fold concentration range) using liquid handling robotics, and then returned to the incubator for assay at a 72 hour time point. Cells were fixed in 4% formaldehyde for 30 minutes and then stained with 1 μmol/L fluorescent nucleic acid stain Syto60 (Invitrogen) for 1 hour. For suspension cell lines, cells were treated with compound immediately following plating, returned to the incubator for a 72 hour time point, then stained with 55 μg/ml Resazurin (Sigma) prepared in Glutathione-free media for 4 hours. Quantization of fluorescent signal intensity was performed using a fluorescent plate reader at excitation and emission wavelengths of 630/695 nm for Syto60, and 535/595 nM for Resazurin. All screening plates are subjected to stringent quality control measures and to assess the quality of our screening a Z-factor score comparing negative and positive control wells is calculated across all screening plates. Effects on cell viability are measured and a curve-fitting algorithm is applied to this raw dataset to derive a multi-parameter description of drug response, including the half maximal inhibitory concentration (IC_{50}) (the concentration that gives a 50% reduction in cell number relative to untreated control wells) and the slope of the dose response curve. Scatter plots of cell line IC_{50} values are provided to allow examination of cell line sensitivity to a drug based on the mutational/copy number status of a cancer gene (*MET*).

The dose response curves were fitted to raw fluorescence intensity values using a bespoke Bayesian sigmoid model. This models acute and partial responses to a drug that fall within the range of experimental screening concentrations. In many instances however, a significant proportion of cell lines will be resistant to a given drug within the range of experimental screening concentrations. The curve-fitting algorithm reports IC-values for these cell lines, which are associated with large confidence intervals. For completeness these values have been reported but they should be interpreted carefully and, before performing further analyses, it may be appropriate

to restrict the IC_{50} value to the maximum screening concentration, or use an alternative output such as AUC.

Genomic analysis of H1993 resistant clones with deep targeted sequencing

Targeted sequencing of the parental H1993 cell line and eight resistant clones was performed on the Illumina HiSeq2500 instrument. Purified DNA was enriched for all exons of the 45 genes from the Qiagen GeneRead Lung v2 panel. Libraries were prepared and indexed using manufacturer's instructions. Raw sequncing data in a FASTQ format were processed and used for analysis of single nucleotide variants (SNVs), indels and copy number assessment as previously described in [2]. Sequencing data in BAM format was submitted to the NCBI's Sequence Read Archive with submission number SUB1059255.

SUPPLEMENTARY TABLES

COMPOUND NAME	SOURCE	CATALOG No.	TARGET
afatinib	AstraZeneca		EGFR1
amuvatinib	Selleck Chemicals	S1244	C-KIT
ASP3026	AstraZeneca		ALK
AZD1208	AstraZeneca		pan-PIM
AZD2014	AstraZeneca		pan-mTOR
AZD5363	AstraZeneca		AKT
AZD6094	AstraZeneca		MET
AZD8055	AstraZeneca		pan-mTOR
AZD8931	AstraZeneca		EGFR1/2/3
BGJ398	AstraZeneca		FGFR1-4
BI-D1870	AstraZeneca		pan-RSK
BMS-777607	Selleck Chemicals	S1561	Tyro3/DTK
bosutinib	AstraZeneca		SRC
cabozantinib	Selleck Chemicals	849217-68-1	C-KIT/vegfr2
canertinib	AstraZeneca		EGFR family
CP-466722	AstraZeneca		ATM
CP-673451	AstraZeneca		PDGFRα/β
CP-724714	AstraZeneca		EGFR2 (HER2)
crenolanib	AstraZeneca		PDGFRα/β
crizotinib	MedKoo	202222	MET/
dacomitinib	AstraZeneca		pan-EGFR
danusertib	Kingston Chemistry	KST-09971170	RET
dasatinib	AstraZeneca		Abl
erlotinib	Maybridge	GK3654	EGFR family
fludarabine	AstraZeneca		stat1
GDC-0941	AstraZeneca		pan-PI3K
GDC-0994	AstraZeneca		ERK1/2
gefitinib	AstraZeneca		EGFR family
JNK Inhibitor IX	Life Chemicals	F0016-0404	JNK
KU-60019	AstraZeneca (KuDOS)		ATM
lapitinib	AstraZeneca		EGFR family
masitinib	Toronto Research Chemicals Inc	M197500	C-KIT
NVP-AEW541	AstraZeneca		IGFR/INSR
palbociclib	Chemietek	CT-PD2991	CDK4/6
PF-04691502	WuXi PharmaTech	LS048/10	mTORC1
PF-4708671	AstraZeneca		p70 S6K1
PF-573228	AstraZeneca		FAK
picolinamide PIMi	AstraZeneca		pan-PIM
PLX4032	AstraZeneca		B-raf (V600E)
quizartinib	AstraZeneca		FLT3
RAD001	Sequoia Research Products	SRP02750e	mTORC1
SCH772984	AstraZeneca		ERK1/2
TAE684	AstraZeneca		ALK
TPCA-1	AstraZeneca		IKK2
WZ4002	Selleck Chemicals	S1173	mEGFR1 (L858R)/(T790M)
SGX-523	Selleck Chemicals	S1112	MET
JNJ-38877605	Selleck Chemicals	S1114	MET
PHA-665752	Selleck Chemicals	S1070	MET

Supplementary Table S1 | Chemical compounds used in this study. All compounds from commerical sources are listed with their suppliers and catalog numbers. All non-catalog items were synthesized by or on behalf of AstraZeneca.

epitope	manufacturer	catalog number	dilution
pMET (Y1234/Y1235)	Cell Signaling Technology	3077	1:1000
pMET (Y1003)	Cell Signaling Technology	3135	1:1000
total MET	Cell Signaling Technology	8198	1:1000
pERK1/2(T202/Y204)	Cell Signaling Technology	4370	1:1000
total ERK1/2	Cell Signaling Technology	4695	1:1000
pAKT (S473)	Cell Signaling Technology	4060	1:1000
total AKT	Cell Signaling Technology	4691	1:1000
pEGFR (Y1068)	Cell Signaling Technology	3777	1:1000
total EGFR	Cell Signaling Technology	2232	1:1000
pErbB3 (Y1289)	Cell Signaling Technology	4791	1:1000
total ErbB3	Cell Signaling Technology	12708	1:1000
cleaved caspase 3	Cell Signaling Technology	9661	1:1000
nucleolin	Santa Cruz Biotechnology, Inc.	sc-8031	1:1000
pMEK(S217/S221)	Cell Signaling Technology	2354	1:1000
total MEK	Cell Signaling Technology	9122	1:1000
pSTAT3 (S727)	Cell Signaling Technology	9136	1:1000
total STAT3	Cell Signaling Technology	9132	1:1000
cMYC	Cell Signaling Technology	5605	1:1000
vinculin	Sigma-Aldrich	V4505	1:10,000
α-tubulin	Sigma-Aldrich	T9026	1:5000
pS6(S235/S236)	Cell Signaling Technology	2211	1:1000
total S6	Cell Signaling Technology	2317	1:1000
actin	Santa Cruz Biotechnology, Inc.	sc-1616-R	1:1000
horse anti-mouse IgG-HRP conjugated 2°	Cell Signaling Technology	7076	1:5000
goat anti-rabbit IgG-HRP conjugated 2°	Cell Signaling Technology	7074	1:5000

Supplementary Table S2 | Antibodies used in this study. All primary antibodies were incubated with membranes overnight at 4°C in Tris-buffered saline-Tween20 (TBST) solution containing 3% (w/v) Fraction-V BSA. Secondary antibodies were incubated for 1-2 hours at room temperature in TBST containing 5% (w/v) non-fat dry milk.

SUPPLEMENTARY REFERENCES

1. Yang W, Soares J, Greninger P, Edelman EJ, Lightfoot H, Forbes S, Bindal N, Beare D, Smith JA, Thompson IR, Ramaswamy S, Futreal PA, Haber DA, et al. Genomics of Drug Sensitivity in Cancer (GDSC): a resource for therapeutic biomarker discovery in cancer cells. Nucleic Acids Res. 2013; 41:D955-61.
2. Eberlein CA, Stetson D, Markovets AA, Al-Kadhimi KJ, Lai Z, Fisher PR, Meador CB, Spitzler P, Ichihara E, Ross SJ, Ahdesmaki MJ, Ahmed A, Ratcliffe LE, et al. Acquired Resistance to the Mutant-Selective EGFR Inhibitor AZD9291 Is Associated with Increased Dependence on RAS Signaling in Preclinical Models. Cancer Res. 2015; 75:2489-500.
